# Scalable Bottom-Up Synthesis of Nanoporous Hexagonal Boron Nitride (*h*-BN) for Large-Area Atomically Thin Ceramic Membranes

**DOI:** 10.1021/acs.nanolett.4c05939

**Published:** 2025-02-14

**Authors:** Andrew E. Naclerio, Peifu Cheng, Saban M. Hus, J. Trey Diulus, Marti Checa, Ivan Vlassiouk, William H. Fissell, Matthew Coupin, Jamie Warner, Liam Collins, Andrei Kolmakov, An-Ping Li, Piran R. Kidambi

**Affiliations:** † Department of Chemical and Biomolecular Engineering, Vanderbilt University, Nashville, Tennessee 37212, United States; ‡ Center for Nanophase Materials Sciences, Oak Ridge National Laboratory, Oak Ridge, Tennessee 37831, United States; § Nanoscale Device Characterization Division, PML, NIST, Gaithersburg, Maryland 20899, United States; ∥ Department of Medicine and Division of Nephrology and Hypertension, Vanderbilt University Medical Center, Nashville, Tennessee 37232, United States; ⊥ Vanderbilt Institute of Nanoscale Sciences and Engineering, Vanderbilt University, Nashville, Tennessee 37212, United States; # Department of Mechanical Engineering, Vanderbilt University, Nashville, Tennessee 37212, United States; ∇ Walker Department of Mechanical Engineering, University of Texas at Austin, Austin, Texas 78712, United States

**Keywords:** hexagonal boron nitride (*h*-BN), chemical vapor deposition (CVD), *h*-BN membranes, ceramic membranes, nanoporous atomically thin membranes, nanopores, defects in *h*-BN

## Abstract

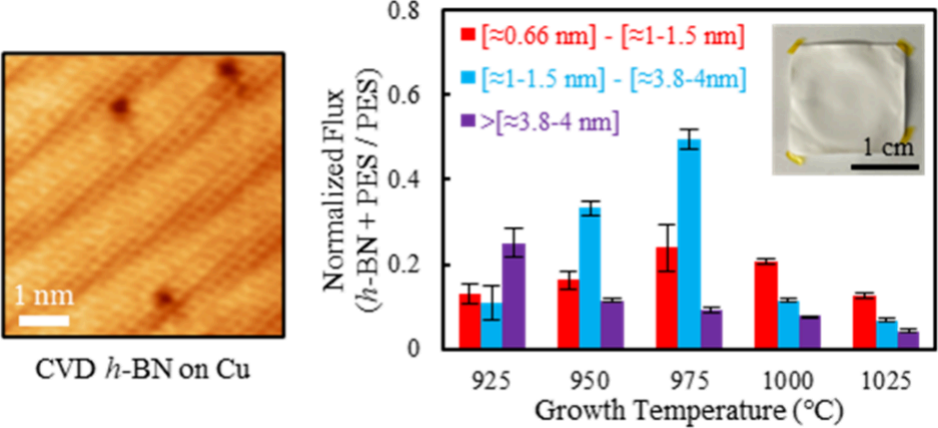

Nanopores embedded within monolayer hexagonal boron nitride (*h*-BN) offer possibilities of creating atomically thin ceramic membranes with unique combinations of high permeance (atomic thinness), high selectivity (via molecular sieving), increased thermal stability, and superior chemical resistance. However, fabricating size-selective nanopores in monolayer *h*-BN via scalable top-down processes remains nontrivial due to its chemical inertness, and characterizing nanopore size distribution over a large area remains extremely challenging. Here, we demonstrate a facile and scalable approach of exploiting the chemical vapor deposition (CVD) process temperature to enable direct incorporation of subnanometer/nanoscale pores into the monolayer *h*-BN lattice, in combination with manufacturing compatible polymer casting to fabricate centimeter-scale nanoporous atomically thin ceramic membranes. We leverage diffusive transport of analytes including size-selective Ficoll sieving to characterize subnanometer-scale and nanoscale defects that manifest as pores in centimeter-scale *h*-BN membranes, overcoming previous limitations in large-area characterization of nanoscale defects in *h-*BN. Our approach opens a new frontier to advance atomically thin membranes to 2D ceramic materials, such as *h*-BN via facile and direct formation of nanopores, for size-selective separations.

Nanoporous atomically thin membranes offer transformative potential across a broad spectrum of applications including ionic/molecular separations, small molecule separation, dialysis, gas separation, proton exchange membranes, isotope separations, among others.^1−7^ Among the different atomically thin 2D materials, monolayer hexagonal boron nitride (*h*-BN) offers a unique combination of atomic thinness, exceptional chemical resistance, remarkable thermal stability, and most importantly, its capacity to support lattice defects that manifest as nanopores in an atomically thin membrane.^8−12^ These properties make monolayer *h*-BN ideally suited for fabricating atomically thin ceramic membranes with both high permeance (due to atomic thinness) and selectivity (via molecular sieving).^1,6^*h*-BN membranes have been explored for applications including DNA translocation, proton exchange^4,13−16^ and isotope separation^3,17^ as well as ionic^18,19^ and molecular separations.

However, introducing precise nanopores into monolayer *h*-BN presents significant challenges. Unlike graphene, where top-down etching techniques such as oxygen plasma and/or chemical etching readily introduce pores via a scalable process over large areas, the chemical inertness of *h*-BN limits pore formation to e-beam^20,21^ and ion irradiation^22^ that are often limited to small areas and low throughput or reactive ion etching techniques^23,24^ or XeF_2_ etching,^25^ necessitating multiple process steps. Another challenge is the absence of a rapid and reliable method for characterizing defects in *h*-BN, preventing an effective feedback loop. For example, while graphene benefits from Raman spectroscopy as a reliable tool for defect analysis, equivalent techniques for *h*-BN currently remain elusive.^26,27^ Current methods for nanoscale *h*-BN defect characterization such as scanning tunnelling microscopy/spectroscopy (STM/S) and scanning transmission electron microscopy (STEM) are expensive, have low throughput, and are impractical for large-scale characterization limiting progress of nanoporous atomically thin *h*-BN membranes.^8,21,28,29^ We note that the ability to probe defects in the subnanometer to nanoscale range over large areas in monolayer *h*-BN is highly relevant to applications beyond atomically thin membranes,^15−17,21,30−35^ e.g., nanoelectronics,^36−46^ neutron detectors,^47−53^ photonics,^41,54−58^ oxidation protection,^59^ semiconductor processing,^60,61^ and others.

Here, we introduce a novel, scalable bottom-up approach to directly incorporate subnanometer-scale and nanoscale pores into monolayer *h*-BN during CVD by precisely tuning the synthesis temperature. While bottom-up synthesis of nanoporous monolayer graphene to fabricate atomically thin membranes exists,^7,62−72^ they remain elusive for *h*-BN. Additionally, we leverage a manufacturing-compatible process that integrates CVD-grown *h*-BN with poly ether sulfone (PES) through phase inversion, resulting in centimeter-scale *h*-BN+PES composite membranes.^63,73^ Further, we use diffusion-based transport experiments,^63,74,75^ including size-selective Ficoll sieving,^76−78^ to characterize nanopore size distribution over large membrane areas. Our results indicate that CVD growth conditions play a critical role; i.e., subnanometer pores are maximized at intermediate temperatures (∼975 °C). This controlled bottom-up nanopore formation, governed by the kinetics of the growth and etching processes, offers a pathway to enable atomically thin *h*-BN membranes. Finally, the *h*-BN membranes demonstrate separation factors as high as ∼97 for KCl (∼0.66 nm) versus lysozyme (∼3.8–4 nm) and ∼43 for l-tryptophan (∼0.7–0.9 nm) versus lysozyme, alongside high permeance values (KCl ∼(2.1–7.4) × 10^–6^ m s^–1^, l-Tr ∼(0.7–3.3) × 10^–6^ m s^–1^, Lz ∼(3.6–20.9) × 10^–8^ m s^–1^) for model nanoscale separations.^79^

## Bottom-up Synthesis of Monolayer *h*-BN via CVD

Atomically thin membrane applications necessitate large-area synthesis of 2D materials, and chemical vapor deposition (CVD) has emerged as the preferred route for scalable and cost-effective high-quality 2D material synthesis,^8,80^ including proof-of-concept demonstrations of manufacturing compatible roll-to-roll approaches.^81−84^ However, 2D materials synthesized via CVD contain intrinsic lattice defects viewed as detrimental to electronic applications.^1,4,6,85^ For membrane applications, defects in the 2D lattice manifest as pores in an atomically thin membrane. Here, we demonstrate direct incorporation of nanopores into the atomically thin *h*-BN lattice during CVD via facile tuning of CVD growth temperature and enable the creation of nanoporous atomically thin ceramic membranes with size-selective transport without the need for postsynthesis top-down pore creation approaches that are challenging for ceramics/*h*-BN. Unlike graphene where Raman spectroscopy offers a measure of defects in the 2D lattice,^26^ no equivalent technique currently exists for probing defects in *h*-BN. Here, we use size-selective transport as a measure of defect sizes in monolayer *h*-BN over centimeter-scale areas by fabricating atomically thin membranes.^86^

**Figure 1:**
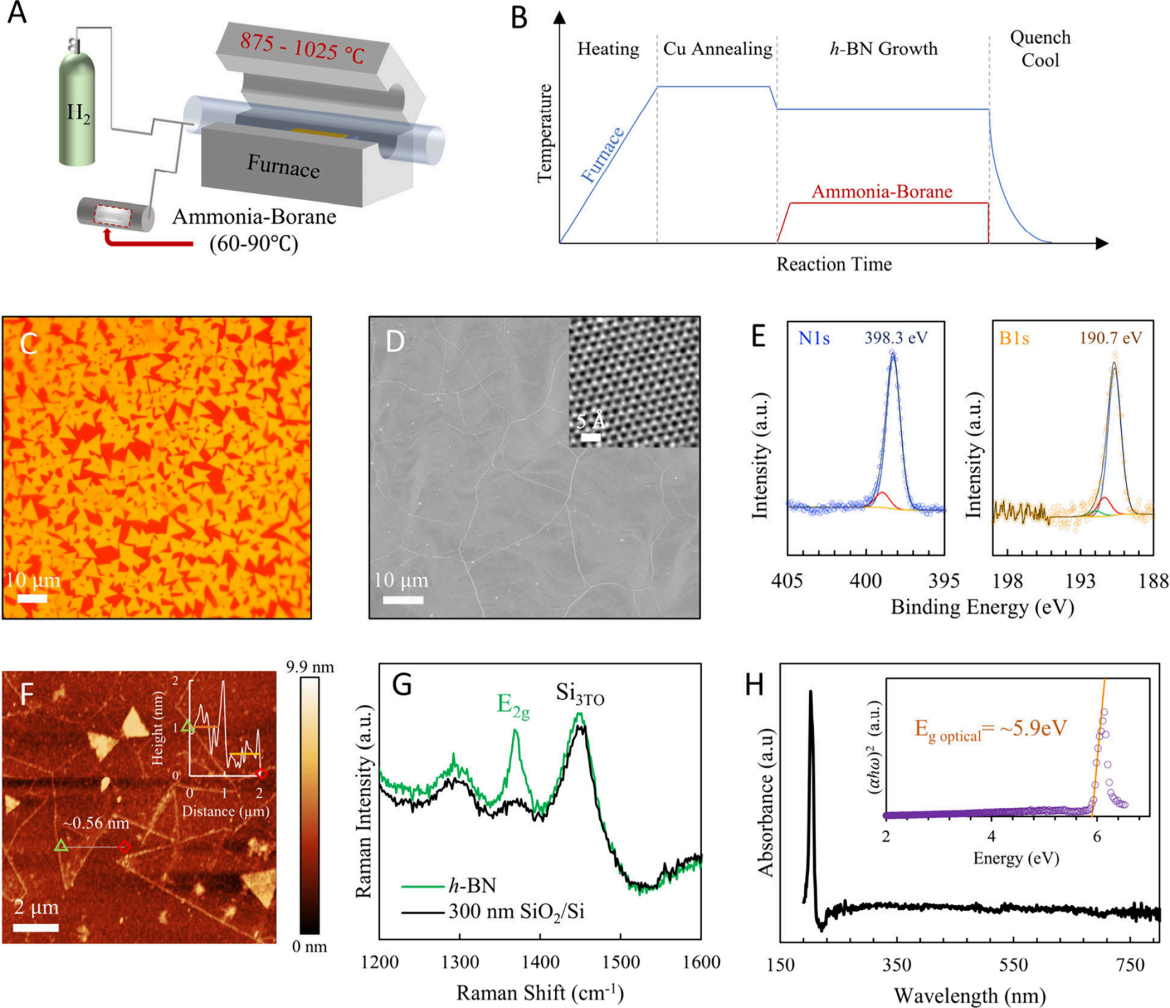
Bottom-up synthesis of monolayer *h*-BN. A) Hot-wall chemical vapor deposition (CVD) reactor setup using ammonia–borane as precursor for *h*-BN synthesis on polycrystalline Cu foil (also see Figure S6). B) Schematic of reactor and precursor chamber temperature profiles during CVD process. C) Exemplar optical image of unmerged monolayer triangular domains of *h*-BN on Cu foil after oxidization of the Cu foil by heating on a hot plate at ∼200 °C. D) SEM image of complete *h-*BN film on Cu, identified via wrinkles in *h*-BN formed due to difference in thermal expansion between *h*-BN and the underlying Cu catalyst. Inset: STEM image of *h*-BN lattice (also see Figure S8). E) XPS spectra of *h*-BN on Cu with characteristic N 1s and B 1s spectra. F) AFM micrograph and line profile of edge of *h*-BN film transferred to 300 nm SiO_2_/Si wafer. The thickness of the film is observed to be ∼0.56 nm, consistent with monolayer *h-*BN. G) Raman spectrum of continuous *h*-BN film transferred to 300 nm SiO_2_/Si wafer shows the characteristic E_2g_ peak. H) UV–vis absorption spectra and the computed Tauc plot and calculated optical bandgap of ∼5.9 eV.^97,104^ All characterizations presented in Figure 1 are performed on *h*-BN grown for 90 min (for continous films and ∼22−25 min for unmerged domains) at 1025 °C with 3.5 mg precursor at 85 °C (see Supporting Information).

**Figure 2:**
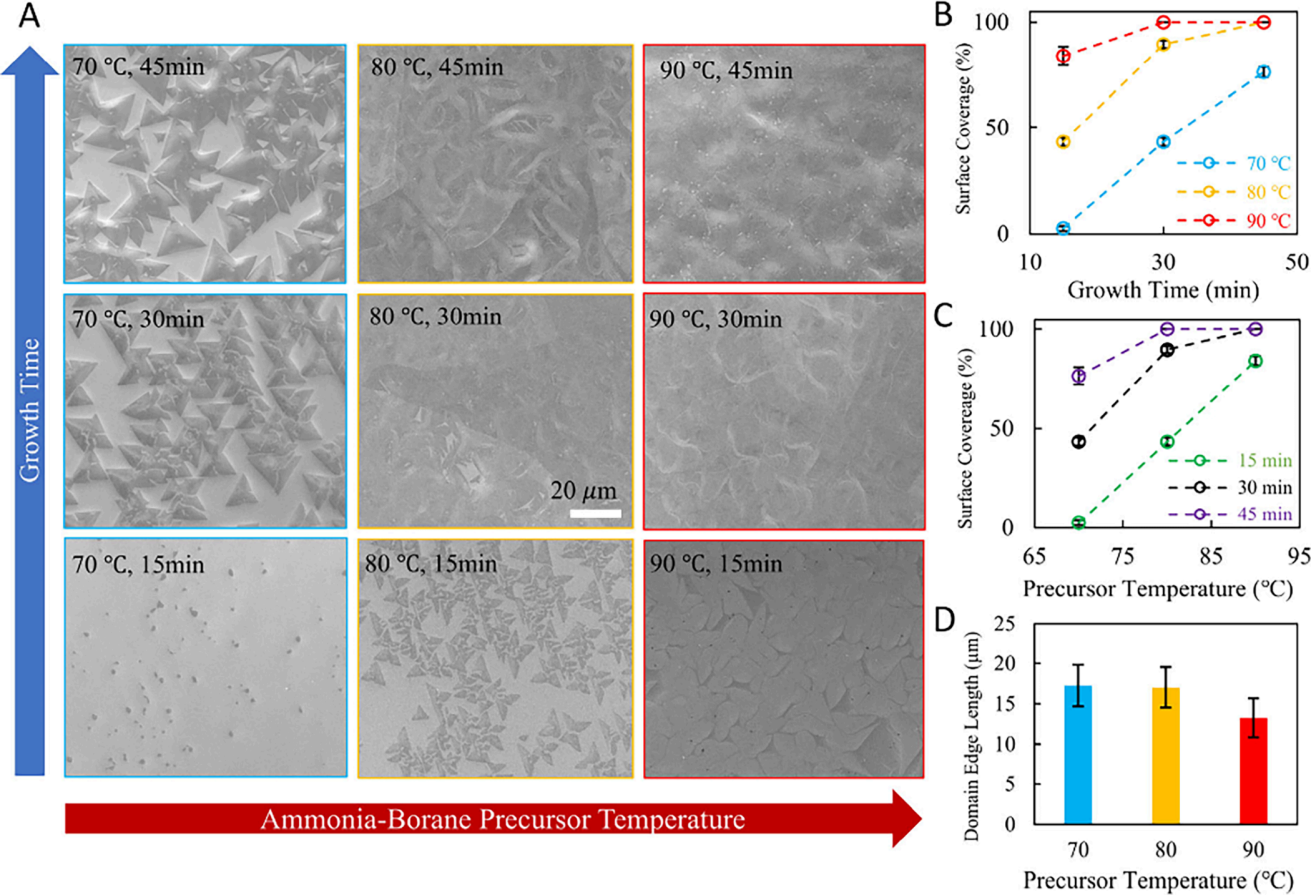
Kinetic control of *h*-BN surface coverage on Cu via CVD growth time and ammonia borane delivery. A) SEM images of *h*-BN (grown at 1025 °C) on Cu foil showing changes in surface coverage as a function of growth time for different temperatures of ammonia borane (∼3 mg) in the side chamber (see Supporting Information). *h*-BN surface coverage on Cu (computed from A using ImageJ software, see methods) as a function of B) growth time and C) ammonia–borane precursor temperature. Error bars indicate one standard deviation. D) Average *h*-BN domain size (edge length of triangular domains) computed before the film converges to a complete layer as a function of ammonia–borane precursor temperature. Error bars indicate one standard deviation.

**Figure 3:**
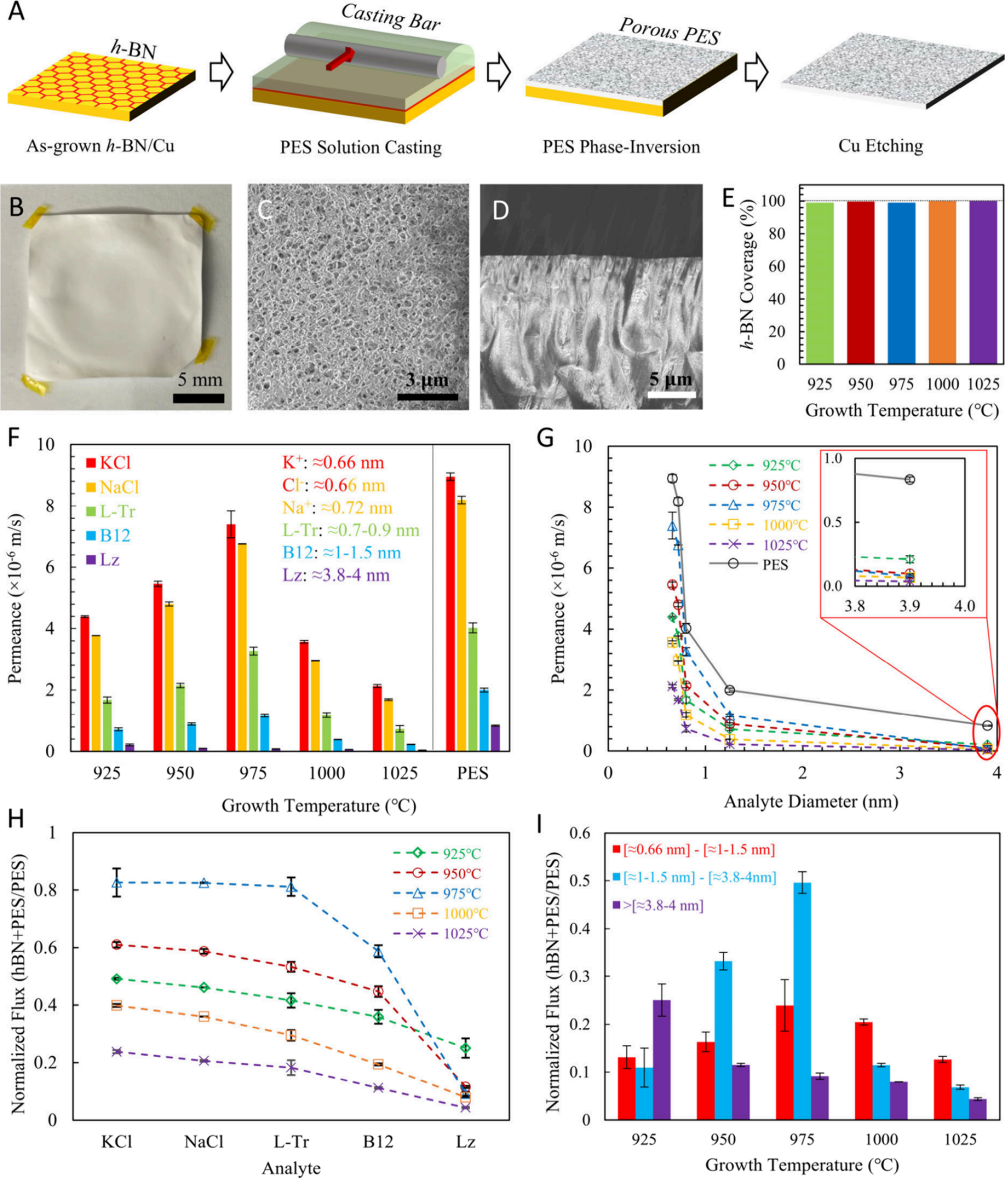
Facile PES casting allows probing intrinsic defects in monolayer *h*-BN manifesting as nanopores in an atomically thin ceramic membrane. A) Schematic of the facile PES casting process using nonsolvent induced phase inversion to form a porous support for *h*-BN.^79,84^ B) Optical image and C) top view SEM image of *h*-BN on PES supports. D) SEM cross sectional image of the PES support layer showing hierarchical porosity, PES pores in the vicinity of *h*-BN form ∼300–500 nm structures that effectively support *h*-BN but branch out to more finger-like structures and widen down the thickness of the membrane, forming a network of interconnected channels.^79,84^ E) Pressure driven ethanol flow through the *h*-BN/PES membranes is measured and coverage is computed by normalizing it to a representative control PES membrane (see Supporting Information). F) Diffusive permeance of KCl (K^+^, Cl^–^ ∼0.66 nm hydrated diameter), NaCl (Na^+^ ∼0.72 nm hydrated diameter), l-tryptophan (l-Tr ∼0.7–0.9 nm), vitamin B12 (B12 ∼1–1.5 nm), and lysozyme (Lz ∼3.8–4 nm) for *h-*BN/PES and PES reference membranes (see methods) provides an indication of nanopore size distribution in atomically thin monolayer *h*-BN over centimeter-scale areas. Error bars indicate one standard deviation. Also see G) for diffusive permeance as a function of analyte size. Inset shows a zoomed in region of the plot. Error bars indicate one standard deviation. H) Normalized flux for the analytes computed by dividing *h*-BN/PES permeance by PES reference permeance. Error bars indicate one standard deviation. I) Normalized flux attributed to defects of different size ranges in centimeter-scale atomically thin *h*-BN membranes. Normalized flux from defects >3.8 nm decrease with increasing growth temperature, while normalized flux from defects in the sub-nm and few-nm range are maximized at intermediate growth temperatures. Error bars indicate one standard deviation. Note we only use *h*-BN grown at >925 °C for membrane fabrication, since 875 °C since results in an incomplete film (Figure S1).

**Figure 4:**
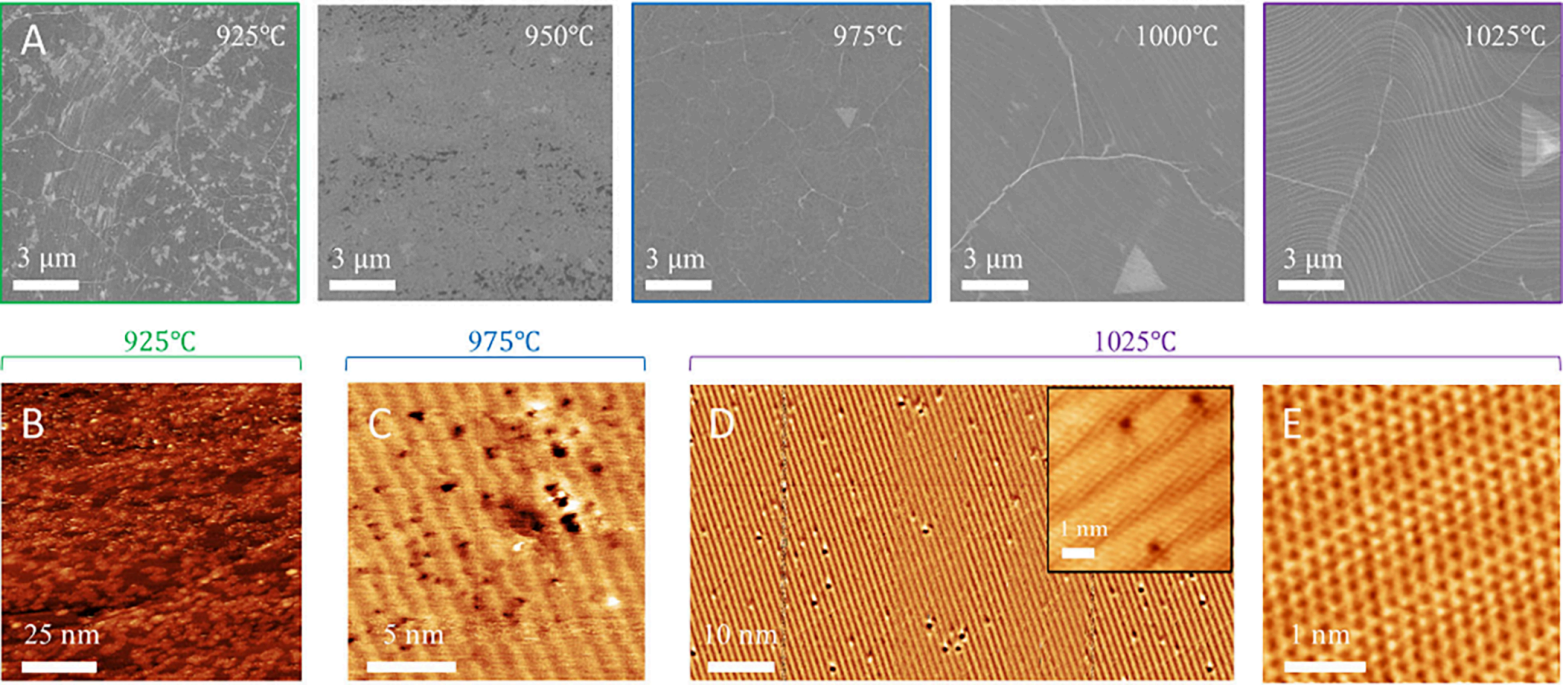
Changes in *h*-BN films with growth temperature. A) SEM images of *h*-BN grown at 925, 950, 975, 1000, and 1025 °C. B) Representative STM image of *h*-BN grown on Cu at 925 °C shows significant coverage with features consistent with surface clusters and atomic resolution STM imaging was nontrivial. C) Representative high-resolution STM images of *h*-BN grown at 975 °C show a high density of subnanometer-scale and nanoscale defects consistent with Figure 3. D) Representative STM images of *h*-BN grown at 1025 °C shows the lowest number of defects indicating the highest quality. Inset: atomic resolution STM image of defects in *h*-BN grown at 1025 °C, while E) shows a pristine region of the *h*-BN lattice on Cu. The line like features in the STM images in C,D, and E are attributed to the Moire patterns from *h*-BN on Cu(100) surface. Images B, C, and D: *V*_S_ = −2 V. Image E: *V*_S_ = −3 mV. Note extracting nanopore density and size distribution from STM by scanning over large areas is not feasible.

**Figure 5:**
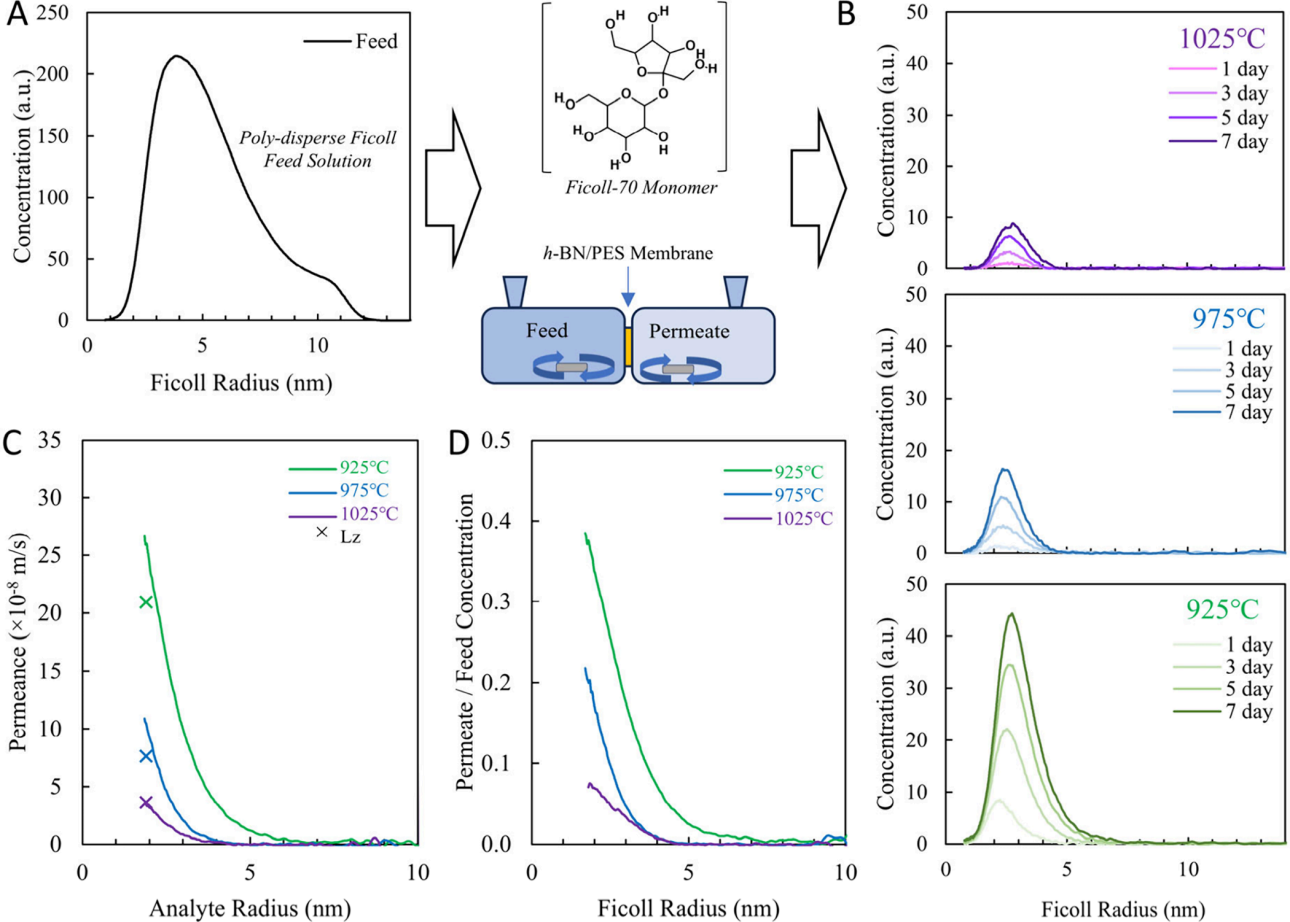
Characterizing size distribution of defects in atomically thin *h*-BN/PES membranes via facile Ficoll diffusion. A) Schematic of Ficoll experiment.^76^ Ficoll concentrations in the feed as a function of Ficoll radius and B) permeate after 1, 3, 5, and 7 days (light to dark hue/shade) for *h*-BN + PES membranes fabricated using *h*-BN synthesized at different growth temperatures. Ficoll permeate shows an increasing concentration of Ficoll over time and larger Ficoll size cutoff for *h*-BN grown at lower growth temperature consistent with diffusive transport in Figure 3I. C) Ficoll permeance as a function of analyte radius shows good agreement and lysozyme diffusion in Figure 3. D) Ratio of Ficoll concentration in permeate after 7 days/Ficoll feed concentration as a function of Ficoll radius.

We use a custom-built hot-wall reactor (Figure 1A and Figure S6) with ammonia–borane precursor and H_2_ gas to synthesize monolayer *h*-BN via CVD (Figure 1B) on polycrystalline copper (Cu) foil catalysts (see methods).^8,80^ Ammonia–borane (precursor for B and N atoms^87,88^) is heated in a side chamber and the products are introduced into the reactor with H_2_ as the carrier gas to the Cu foil catalyst.^87,88^ The Cu foil catalyzes monolayer *h*-BN via precursor dissociation, nucleation of *h*-BN domains (Figure 1C), and subsequent growth of the domains to merge forming a polycrystalline film with increasing CVD growth time (Figure 1D, Figure 2A). The triangular shape of the nuclei is attributed to the higher stability of N terminated edges.^86,87^ The side chamber temperature (∼70–90 °C) allows for control over precursor delivery to the CVD reactor^87,88^ (Figure 2) and the CVD reactor temperature (∼875–1025 °C) allows for control over film morphology (see Figure S1) and quality (Figures 3–5) as discussed further below. We only use *h*-BN grown at >925 °C for membrane fabrication (Figure 3), since growth at 875 °C results in an incomplete film (Figure S1).

*h*-BN nuclei on Cu foil appear as individual unmerged triangular domains that are readily visible via optical microscopy (Figure 1C) after selective oxidization of the uncovered Cu foil surface by heating it on a hot plate in ambient environment.^89−92^ SEM images further confirm the triangular *h*-BN nuclei (Figure 2A images on the left column) on Cu foil as well as continuous *h*-BN films formed with increasing growth time (Figure 1D, Figure 2A images on the right column) and identified via with characteristic features such as wrinkles formed due to the difference in thermal expansion between *h*-BN and the underlying Cu catalyst upon cooling to ambient temperature.^93^ Additionally, secondary and ad-layers are also seen as smaller triangles along with wrinkles in the continuous *h*-BN films (Figures 1D and 2A, Figure S1).

SEM images (Figure 2A) show *h*-BN surface coverage (Figure 2B) increases with time for all side chamber temperatures (∼70–90 °C) indicating precursor delivery throughout the growth period.^87,88^ The side chamber temperature also influences the rate of *h*-BN growth on Cu (Figure 2C); i.e., complete surface coverage (continuous film) is achieved within 30 min for side chamber temperatures ∼90 °C, while temperature ∼70 °C yields incomplete films even after 45 min (Figure 2A).^87,88^ These observations indicate that the CVD process is in a kinetic regime limited by precursor supply.^80,94−98^ Finally, *h-*BN domain sizes (triangular edge length) measured just before full film coalescence (Figure 2D) decrease from ∼17 to ∼13 μm upon increasing side chamber temperature from 80 to 90 °C (Figure 2D), indicating higher nucleation density with increased precursor delivery at 90 °C.^98^

X-ray photoelectron spectroscopy of the *h*-BN films on Cu shows characteristic peaks in the core level N 1s (∼398.3 eV) and B 1s (∼190.7 eV) spectra (Figure 1E) with a B:N ratio ∼1 (Figure S2), confirming the elemental composition and stoichiometric balance of B and N atoms, respectively.^80,99^ Atomic force microscopy of an *h*-BN unmerged film transferred to 300 nm SiO_2_/Si wafer shows film thickness ∼0.56 nm, consistent with monolayer *h*-BN^93,100^ (Figure 1F), and Raman spectra show the characteristic E_2g_ peak ∼1372 cm^–1^ for *h*-BN (Figure 1G).^27,101^ Finally, the UV–vis absorption spectrum (Figure 1H) shows a strong absorbance peak at ∼202 nm and the corresponding Tauc plot indicates an optical bandgap of ∼5.9 eV, also consistent with monolayer *h*-BN.^54,93,97,102−105^ Finally, atomic resolution scanning tunneling microscopy (STM) and scanning transmission electron microscopy images (STEM) images show a hexagonal honeycomb mesh and further confirm the high crystallinity of the *h*-BN films (Figure 4E and inset of Figure 1D).

## Nanoporous Atomically Thin *h*-BN Membrane Fabrication and Size-Selective Transport

Having confirmed atomically thin *h*-BN films have been synthesized via CVD on Cu, we proceed to fabricate centimeter-scale nanoporous atomically thin ceramic membranes via facile and scalable polymer casting (Figure 3A).^79,84^ A thin layer of polyether sulfone (PES) solution is cast onto *h*-BN on Cu and the resulting stack (PES|*h*-BN|Cu foil) is immersed immediately in a water bath to induce phase inversion of PES (Figure 3A) converting it into porous supports for *h*-BN.^79,84^ Subsequent removal of the Cu foil via etching results in large-area *h*-BN transferred to porous PES supports, forming atomically thin ceramic membranes (see the optical image in Figure 3B). SEM images (Figure 3C—top view, Figure 3D—cross section, and Figure S3) show 300–500 nm pores in the PES in the vicinity of *h*-BN effectively supporting the atomically thin monolayer and branch out to more finger-like structures further below, forming a hierarchical network of interconnected channels (Figure 3C,D, Figure S3) within the ∼50 μm thick PES supports (Figure S3A).^79,84^ The insulating nature of *h*-BN and PES presents challenges with SEM imaging due to charging, but adlayers are visible upon careful observation (Figure S3C,D), indicating the presence of the *h*-BN film on the PES supports (PES surface porosity ∼50%, Imagej analysis of SEM images).

Pressure driven transport of ethanol confirms *h*-BN is successfully transferred to PES supports and relatively free of large tears.^75,85^ Notably, compared to the control membrane, i.e., PES support (see methods), *h*-BN+PES effectively blocks >99% of pressure-driven ethanol flow (Figure 3E). Next, we proceed to measure and compare molecular diffusion through nanopores in *h*-BN to i) demonstrate molecular sieving though nanopores in *h*-BN membranes and ii) use diffusive transport to characterize nanopore sizes in *h*-BN as a function of synthesis temperature (Figure 3F–I, Figure 5).

Initially, we probe diffusive transport of single species using model analytes from subnanometer to nanometer length scales, i.e., KCl (hydrated diameter K^+^ ∼0.66 nm, Cl^–^ ∼0.66 nm), NaCl (hydrated diameter Na^+^ ∼0.72 nm, Cl^–^ ∼0.66 nm), l-tryptophan (l-Tr ∼0.7–0.9 nm), vitamin B12 (B12 ∼1–1.5 nm), and lysozyme (Lz ∼3.8–4 nm).^74,75,79,84^ Notably, the addition of *h*-BN significantly reduces the diffusive permeance of all species compared to PES controls without *h*-BN (Figure 3F), indicating the observed transport resistance originates for monolayer *h*-BN. Further, the diffusive permeances though *h*-BN+PES and PES control membranes show a decreasing trend with increasing analyte size consistent with lower diffusivity for larger species (Figure 3G). The effectiveness of the PES control membranes is observed via the selectivity (ratio of permeance) of the probed analytes being similar to i) polycarbonate track etched membranes with straight channel ∼200 nm pores that do not overlap and ii) ratio of diffusivity of analytes in free solution (Figure S4).^106−110^

To deconvolute transport from solution diffusivity, we use the permeance ratio of the *h*-BN+PES membrane to the PES control membranes, i.e., normalized flux (Figure 3H). We observe a reduction in normalized flux with increasing analyte size for the *h*-BN membranes, indicating the presence of nanopores in the ∼0.66–4 nm size range (Figure 3I); e.g., *h*-BN synthesized at 1025 °C exhibits normalized flux of KCl ∼23.8%, NaCl ∼20.6%, l-Tr ∼18.2%, B12 ∼11.1% and Lz ∼4.3%. Interestingly, a reduction in CVD temperatures results in significantly higher normalized flux; e.g., *h*-BN synthesized at 975 °C shows normalized flux of KCl ∼82.6%, NaCl ∼82.4%, l-Tr ∼81.1%, B12 ∼58.7% and Lz ∼9.1% (Figure 3I), indicating facile incorporation of nanopores into the *h*-BN lattice via a reduction in CVD temperature. Further, the normalized flux of Lz consistently increases as the *h*-BN growth temperature decreases, i.e. Lz ∼4.3% (∼1025 °C), ∼7.9% (∼1000 °C), ∼9.1% (∼975 °C), ∼11% (∼950 °C), ∼25% (∼925 °C), respectively, indicating an increase in defects >3.8–4 nm with lower *h*-BN growth temperatures. However, the permeance of smaller species do not particularly follow this trend; e.g., *h*-BN grown at 975 °C shows the highest normalized flux for KCl, NaCl, l-Tr, and B12 with higher and lower growth temperature decreasing the normalized flux. Finally, we note that the entire process from *h*-BN synthesis to membrane fabrication is reproducible across multiple samples (Figure S7).

The difference in normalized flux of the different analytes can be used to aid visualization of the distribution of nanopore sizes in the atomically thin *h*-BN membrane (Figure 3I). For example, the normalized flux associated with defect in the ∼0.66–1.5 nm size range can be obtained by subtracting the normalized flux of B12 (∼1–1.5 nm) from the flux of KCl (∼0.66 nm). Similarly, the difference between normalized flux of Lz (∼3.8–4 nm) and B12 represents defects in the ∼1.5–4 nm size range (Figure 3I). Hence, diffusive transport indicates the highest fraction of defects in the subnanometer size range for *h*-BN grown at ≥1000 °C, the ∼1.5–4 nm range for *h*-BN grown at intermediate temperatures of ∼950 °C and ∼975 °C, and ≥3.8–4 nm for *h*-BN grown at ∼925 °C.^79,80,111^

These observations of diffusive transport are further supported by atomic resolution STM and SEM images of the as-synthesized *h*-BN directly on Cu for different synthesis temperatures (Figure 4, Figure S1) avoiding convolution from transfers processes that leave residues necessitating subsequent aggressive cleaning processes.^7,75,111^ Representative STM images of films grown at 1025 °C shows the hexagonal *h*-BN lattice and a low density of subnanometer defects (Figure 4). A significant increase in number and size of defects is observed upon reducing growth temperature to ∼975 °C in agreement with transport measurements (Figure 3). An analysis of the representative STM images (while acknowledging limitations of STM to small scan areas) indicates defect density ∼6.8 × 10^12^ cm^–2^ for 975 °C and ∼2.4 × 10^12^ cm^–2^ for 1025 °C, respectively.

For temperatures ≤925 °C a distinct change in film morphology is seen (Figure 4 and Figure S1) compared to the higher growth temperatures and STM imaging was nontrivial with an extremely rough surface. However, XPS indicates stochiometric balance of B:N ∼ 1 is maintained for all synthesis temperatures ∼875–1025 °C (Figure S2). While the exact mechanisms of nanopores formation remain an active area of research, we hypothesize it results from the complex interplay of several synergistic as well as competing *h*-BN growth steps including precursor dissociation on the Cu surface to form active species, diffusion of active species on the Cu surface, reaction rate to form the *h*-BN film, incorporation of elemental species into the Cu bulk and solubility, desorption from the Cu surface, and H_2_ etching during growth that can all change with CVD temperature.^80,87,97,112−114^ Notably, a reduction in CVD temperature could potentially change the rate-determining step in the *h*-BN growth process (due to differences in activation energies and frequency factors for each of the different reaction rates involved) allowing for formation of defects that manifest as nanopores. Taken together, a reduction in CVD growth temperature enables straightforward synthesis of nanoporous atomically thin *h*-BN. Remarkably, the *h*-BN + PES membranes fabricated using *h*-BN synthesized ∼975 °C shows selectivity (ratio of permeance) for KCl/Lz ∼97 and l-Tr/Lz ∼43 (Figure S5). To the best of our knowledge, our experiments represent the first demonstration of fully functional centimeter-scale nanoporous atomically thin *h*-BN membranes for nanoscale separations.

While the single-analyte diffusion measurements allowed probing of defects in ∼0.66–4 nm size rage in centimeter-scale atomically thin *h*-BN membranes (Figure 3), characterizing size distribution of defects over a wider size range and with a continuum of analyte sizes could enable advances in *h*-BN defect characterization for membrane as well as other applications.

Using diffusive transport of FITC-labeled Ficoll (branched polymer of sucrose and epichlorohydrin) as feed, we probe defects in the ∼2–24 nm size range (1–12 nm radius) via size-exclusion chromatography of the permeate diffusing through the *h*-BN + PES membranes (Figure 5A) fabricated using *h*-BN synthesized at different growth temperatures.^76−78^Figure 5B shows an increasing concentration of Ficoll over time from 1 to 7 days in the permeate with *h-*BN grown at high temperatures allowing for the least Ficoll transport throughout the size range, while decreasing the *h*-BN growth temperature results in more Ficoll transport (Figure 5B–D), consistent with diffusive transport experiments (Figure 3I). Notably, the size cutoff of 925 °C *h*-BN+PES membranes is ∼7 nm analyte radius, whereas *h*-BN grown at 975 and 925 °C exhbits a size cutoff ∼5 nm analyte radius (Figure 5C,D). Finally, a comparison of Ficoll transport of similar size to Lz (∼2 nm radius) with diffusive transport measurements of Lz (Figure 3) shows good agreement. Taken together, our Ficoll experiments successfully characterize size distribution (ranges ∼2–24 nm) of nanopores/defects in centimeter-scale atomically thin *h*-BN membranes in a resource efficient manner that has previously remained elusive.

In summary, we demonstrate a facile and scalable method for fabricating centimeter-scale atomically thin ceramic membranes via bottom-up synthesis and polymer casting. A reduction in CVD temperature allows for direct incorporation of subnanometer-scale and nanoscale pores into the *h*-BN lattice during growth, alleviating the need for postsynthesis top-down pore creation approaches that remain challenging and increase costs and process complexity. Using a manufacturing compatible polymer casting and phase inversion approach to form porous PES supports for the nanoporous *h*-BN, we demonstrate centimeter-scale atomically thin ceramic membranes. We characterize the nanopores over large areas in atomically thin *h*-BN via diffusive-transport of analytes and size-selective Ficoll sieving to measure size distributions. The resulting *h*-BN membranes show selectivity of ∼97 for KCl/Lz and ∼43 for l-Tr/Lz, alongside significantly high permeance, and present potential for applications including protein desalting, dialysis, and small-molecule separations. Our work provides foundational approaches to enable atomically thin ceramic membranes, unlocking the potential of materials like *h*-BN for nanoscale separations including in potentially harsh environments, i.e., highly acidic/basic as well as organic solvents by leveraging appropriate support materials.

## Supporting Information Available

Experimental methods, additional SEM and XPS characterization of as-grown *h*-BN, additional SEM characterization of PES support, and additional plots on selectivity performance of *h*-BN/PES membrane, reproducibility, picture of the CVD reactor, and STEM images (PDF)

## References

[ref1] Prozorovska L.; Kidambi P. R. State-of-the-Art and Future Prospects for Atomically Thin Membranes from 2D Materials. Adv. Mater. 2018, 30 (52), 1801179 10.1002/adma.201801179. 30085371

[ref2] Kidambi P. R.; Jang D.; Idrobo J.-C.; Boutilier M. S. H.; Wang L.; Kong J.; Karnik R. Nanoporous Atomically Thin Graphene Membranes for Desalting and Dialysis Applications. Adv. Mater. 2017, 29 (33), 1700277 10.1002/adma.201700277. 28656721

[ref3] Lozada-Hidalgo M.; Hu S.; Marshall O.; Mishchenko A.; Grigorenko A. N.; Dryfe R. A. W.; Radha B.; Grigorieva I. V.; Geim A. K. Sieving Hydrogen Isotopes through Two-Dimensional Crystals. Science 2016, 351, 68–70. 10.1126/science.aac9726. 26721995

[ref4] Kidambi P. R.; Chaturvedi P.; Moehring N. K. Subatomic Species Transport through Atomically Thin Membranes: Present and Future Applications. 2021 1979, 374 (6568), eabd7687 10.1126/science.abd7687. 34735245

[ref5] Koenig S. P.; Wang L.; Pellegrino J.; Bunch J. S. Selective Molecular Sieving through Porous Graphene. Nat. Nanotechnol 2012, 7 (11), 728–732. 10.1038/nnano.2012.162. 23042491

[ref6] Wang L.; Boutilier M. S. H.; Kidambi P. R.; Jang D.; Hadjiconstantinou N. G.; Karnik R. Fundamental Transport Mechanisms, Fabrication and Potential Applications of Nanoporous Atomically Thin Membranes. Nat. Nanotechnol 2017, 12 (6), 509–522. 10.1038/nnano.2017.72. 28584292

[ref7] Moehring N. K.; Chaturvedi P.; Cheng P.; Ko W.; Li A.-P.; Boutilier M. S. H.; Kidambi P. R. Kinetic Control of Angstrom-Scale Porosity in 2D Lattices for Direct Scalable Synthesis of Atomically Thin Proton Exchange Membranes. ACS Nano 2022, 16 (10), 16003–16018. 10.1021/acsnano.2c03730. 36201748

[ref8] Naclerio A. E.; Kidambi P. R. A Review of Scalable Hexagonal Boron Nitride (H-BN) Synthesis for Present and Future Applications. Adv. Mater. 2023, 35 (6), 2207374 10.1002/adma.202207374. 36329667

[ref9] Wang L.; Wu B.; Jiang L.; Chen J.; Li Y.; Guo W.; Hu P.; Liu Y. Growth and Etching of Monolayer Hexagonal Boron Nitride. Adv. Mater. 2015, 27 (33), 4858–4864. 10.1002/adma.201501166. 26183904

[ref10] Kostoglou N.; Polychronopoulou K.; Rebholz C. Thermal and Chemical Stability of Hexagonal Boron Nitride (h-BN) Nanoplatelets. Vacuum 2015, 112, 42–45. 10.1016/j.vacuum.2014.11.009.

[ref11] Cretu O.; Ishizuka A.; Yanagisawa K.; Ishizuka K.; Kimoto K. Atomic-Scale Electrical Field Mapping of Hexagonal Boron Nitride Defects. ACS Nano 2021, 15 (3), 5316–5321. 10.1021/acsnano.0c10849. 33577281

[ref12] Vlassiouk I.; Smirnov S.; Puretzky A.; Olunloyo O.; Geohegan D. B.; Dyck O.; Lupini A. R.; Unocic R. R.; Meyer III H. M.; Xiao K. Armor for Steel: Facile Synthesis of Hexagonal Boron Nitride Films on Various Substrates. Adv. Mater. Interfaces 2024, 11 (1), 2300704 10.1002/admi.202470001.

[ref13] Hu S.; Lozada-Hidalgo M.; Wang F. C.; Mishchenko A.; Schedin F.; Nair R. R.; Hill E. W.; Boukhvalov D. W.; Katsnelson M. I.; Dryfe R. A. W.; Grigorieva I. V.; Wu H. A.; Geim A. K. Proton Transport through One-Atom-Thick Crystals. Nature 2014, 516 (7530), 227–230. 10.1038/nature14015. 25470058

[ref14] Yoon S. I.; Seo D.-J.; Kim G.; Kim M.; Jung C.-Y.; Yoon Y.-G.; Joo S. H.; Kim T.-Y.; Shin H. S. AA′-Stacked Trilayer Hexagonal Boron Nitride Membrane for Proton Exchange Membrane Fuel Cells. ACS Nano 2018, 12 (11), 10764–10771. 10.1021/acsnano.8b06268. 30335961

[ref15] Liu S.; Lu B.; Zhao Q.; Li J.; Gao T.; Chen Y.; Zhang Y.; Liu Z.; Fan Z.; Yang F.; You L.; Yu D. Boron Nitride Nanopores: Highly Sensitive DNA Single-Molecule Detectors. Adv. Mater. 2013, 25 (33), 4549–4554. 10.1002/adma.201301336. 23775629

[ref16] Zhou Z.; Hu Y.; Wang H.; Xu Z.; Wang W.; Bai X.; Shan X.; Lu X. DNA Translocation through Hydrophilic Nanopore in Hexagonal Boron Nitride. Sci. Rep 2013, 3 (1), 3287 10.1038/srep03287. 24256703 PMC3836030

[ref17] Darvish Ganji M.; Dodangeh R. Hydrogen Purification Performance of a Nanoporous Hexagonal Boron Nitride Membrane: Molecular Dynamics and First-Principle Simulations. Phys. Chem. Chem. Phys. 2017, 19 (19), 12032–12044. 10.1039/C7CP01665D. 28443917

[ref18] Cun H.; Iannuzzi M.; Hemmi A.; Osterwalder J.; Greber T. Two-Nanometer Voids in Single-Layer Hexagonal Boron Nitride: Formation via the “Can-Opener” Effect and Annihilation by Self-Healing. ACS Nano 2014, 8 (7), 7423–7431. 10.1021/nn502645w. 24937360

[ref19] Caglar M.; Silkina I.; Brown B. T.; Thorneywork A. L.; Burton O. J.; Babenko V.; Gilbert S. M.; Zettl A.; Hofmann S.; Keyser U. F. Tunable Anion-Selective Transport through Monolayer Graphene and Hexagonal Boron Nitride. ACS Nano 2020, 14 (3), 2729–2738. 10.1021/acsnano.9b08168. 31891480 PMC7098055

[ref20] Elbadawi C.; Tran T. T.; Kolíbal M.; Šikola T.; Scott J.; Cai Q.; Li L. H.; Taniguchi T.; Watanabe K.; Toth M.; Aharonovich I.; Lobo C. Electron Beam Directed Etching of Hexagonal Boron Nitride. Nanoscale 2016, 8 (36), 16182–16186. 10.1039/C6NR04959A. 27603125

[ref21] Gilbert S. M.; Dunn G.; Azizi A.; Pham T.; Shevitski B.; Dimitrov E.; Liu S.; Aloni S.; Zettl A. Fabrication of Subnanometer-Precision Nanopores in Hexagonal Boron Nitride. Sci. Rep 2017, 7 (1), 15096 10.1038/s41598-017-12684-x. 29118413 PMC5678191

[ref22] Kozubek R.; Ernst P.; Herbig C.; Michely T.; Schleberger M. Fabrication of Defective Single Layers of Hexagonal Boron Nitride on Various Supports for Potential Applications in Catalysis and DNA Sequencing. ACS Appl. Nano Mater. 2018, 1 (8), 3765–3773. 10.1021/acsanm.8b00903.

[ref23] Grenadier S.; Li J.; Lin J.; Jiang H. Dry Etching Techniques for Active Devices Based on Hexagonal Boron Nitride Epilayers. Journal of Vacuum Science & Technology A 2013, 31 (6), 61517 10.1116/1.4826363.

[ref24] Danielsen D. R.; Lyksborg-Andersen A.; Nielsen K. E. S.; Jessen B. S.; Booth T. J.; Doan M.-H.; Zhou Y.; Bøggild P.; Gammelgaard L. Super-Resolution Nanolithography of Two-Dimensional Materials by Anisotropic Etching. ACS Appl. Mater. Interfaces 2021, 13 (35), 41886–41894. 10.1021/acsami.1c09923. 34431654

[ref25] Son J.; Kwon J.; Kim S.; Lv Y.; Yu J.; Lee J.-Y.; Ryu H.; Watanabe K.; Taniguchi T.; Garrido-Menacho R.; Mason N.; Ertekin E.; Huang P. Y.; Lee G.-H.; M van der Zande A. Atomically Precise Graphene Etch Stops for Three Dimensional Integrated Systems from Two Dimensional Material Heterostructures. Nat. Commun. 2018, 9 (1), 3988 10.1038/s41467-018-06524-3. 30266948 PMC6162276

[ref26] Ferrari A. C.; Basko D. M. Raman Spectroscopy as a Versatile Tool for Studying the Properties of Graphene. Nat. Nanotechnol 2013, 8 (4), 235–246. 10.1038/nnano.2013.46. 23552117

[ref27] Gorbachev R. V.; Riaz I.; Nair R. R.; Jalil R.; Britnell L.; Belle B. D.; Hill E. W.; Novoselov K. S.; Watanabe K.; Taniguchi T.; Geim A. K.; Blake P. Hunting for Monolayer Boron Nitride: Optical and Raman Signatures. Small 2011, 7 (4), 465–468. 10.1002/smll.201001628. 21360804

[ref28] Ryu G. H.; Park H. J.; Ryou J.; Park J.; Lee J.; Kim G.; Shin H. S.; Bielawski C. W.; Ruoff R. S.; Hong S.; Lee Z. Atomic-Scale Dynamics of Triangular Hole Growth in Monolayer Hexagonal Boron Nitride under Electron Irradiation. Nanoscale 2015, 7 (24), 10600–10605. 10.1039/C5NR01473E. 25960354

[ref29] Auwärter W. Hexagonal Boron Nitride Monolayers on Metal Supports: Versatile Templates for Atoms, Molecules and Nanostructures. Surf. Sci. Rep 2019, 74 (1), 1–95. 10.1016/j.surfrep.2018.10.001.

[ref30] Chen C.; Wang J.; Liu D.; Yang C.; Liu Y.; Ruoff R. S.; Lei W. Functionalized Boron Nitride Membranes with Ultrafast Solvent Transport Performance for Molecular Separation. Nat. Commun. 2018, 9 (1), 1902 10.1038/s41467-018-04294-6. 29765025 PMC5954095

[ref31] Azamat J.; Khataee A.; Joo S. W. Molecular Dynamics Simulations of Trihalomethanes Removal from Water Using Boron Nitride Nanosheets. J. Mol. Model 2016, 22 (4), 82 10.1007/s00894-016-2939-7. 26983611

[ref32] Garnier L.; Szymczyk A.; Malfreyt P.; Ghoufi A. Physics behind Water Transport through Nanoporous Boron Nitride and Graphene. J. Phys. Chem. Lett. 2016, 7 (17), 3371–3376. 10.1021/acs.jpclett.6b01365. 27504857

[ref33] Zhang Y.; Shi Q.; Liu Y.; Wang Y.; Meng Z.; Xiao C.; Deng K.; Rao D.; Lu R. Hexagonal Boron Nitride with Designed Nanopores as a High-Efficiency Membrane for Separating Gaseous Hydrogen from Methane. J. Phys. Chem. C 2015, 119 (34), 19826–19831. 10.1021/acs.jpcc.5b04918.

[ref34] Gao H.; Shi Q.; Rao D.; Zhang Y.; Su J.; Liu Y.; Wang Y.; Deng K.; Lu R. Rational Design and Strain Engineering of Nanoporous Boron Nitride Nanosheet Membranes for Water Desalination. J. Phys. Chem. C 2017, 121 (40), 22105–22113. 10.1021/acs.jpcc.7b06480.

[ref35] Weber M.; Koonkaew B.; Balme S.; Utke I.; Picaud F.; Iatsunskyi I.; Coy E.; Miele P.; Bechelany M. Boron Nitride Nanoporous Membranes with High Surface Charge by Atomic Layer Deposition. ACS Appl. Mater. Interfaces 2017, 9 (19), 16669–16678. 10.1021/acsami.7b02883. 28463495

[ref36] Lindsay L.; Broido D. A. Theory of Thermal Transport in Multilayer Hexagonal Boron Nitride and Nanotubes. Phys. Rev. B 2012, 85 (3), 35436 10.1103/PhysRevB.85.035436.

[ref37] Gannett W.; Regan W.; Watanabe K.; Taniguchi T.; Crommie M. F.; Zettl A. Boron Nitride Substrates for High Mobility Chemical Vapor Deposited Graphene. Appl. Phys. Lett. 2011, 98 (24), 242105 10.1063/1.3599708.

[ref38] Majety S.; Doan T. C.; Li J.; Lin J. Y.; Jiang H. X. Electrical Transport Properties of Si-Doped Hexagonal Boron Nitride Epilayers. AIP Adv. 2013, 3 (12), 122116 10.1063/1.4860949.

[ref39] Lindsay L.; Broido D. A. Enhanced Thermal Conductivity and Isotope Effect in Single-Layer Hexagonal Boron Nitride. Phys. Rev. B 2011, 84 (15), 155421 10.1103/PhysRevB.84.155421.

[ref40] Giovannetti G.; Khomyakov P. A.; Brocks G.; Kelly P. J.; van den Brink J. Substrate-Induced Band Gap in Graphene on Hexagonal Boron Nitride: Ab Initio Density Functional Calculations. Phys. Rev. B 2007, 76 (7), 73103 10.1103/PhysRevB.76.073103.

[ref41] Cassabois G.; Valvin P.; Gil B. Hexagonal Boron Nitride Is an Indirect Bandgap Semiconductor. Nat. Photonics 2016, 10, 262 10.1038/nphoton.2015.277.

[ref42] Dean C. R.; Young A. F.; Meric I.; Lee C.; Wang L.; Sorgenfrei S.; Watanabe K.; Taniguchi T.; Kim P.; Shepard K. L.; Hone J. Boron Nitride Substrates for High-Quality Graphene Electronics. Nat. Nanotechnol 2010, 5, 722 10.1038/nnano.2010.172. 20729834

[ref43] Lee G.-H.; Yu Y.-J.; Lee C.; Dean C.; Shepard K. L.; Kim P.; Hone J. Electron Tunneling through Atomically Flat and Ultrathin Hexagonal Boron Nitride. Appl. Phys. Lett. 2011, 99 (24), 243114 10.1063/1.3662043.

[ref44] Piquemal-Banci M.; Galceran R.; Caneva S.; Martin M.-B.; Weatherup R. S.; Kidambi P. R.; Bouzehouane K.; Xavier S.; Anane A.; Petroff F.; Fert A.; Robertson J.; Hofmann S.; Dlubak B.; Seneor P. Magnetic Tunnel Junctions with Monolayer Hexagonal Boron Nitride Tunnel Barriers. Appl. Phys. Lett. 2016, 108 (10), 102404 10.1063/1.4943516.

[ref45] Kamalakar M. V.; Dankert A.; Bergsten J.; Ive T.; Dash S. P. Enhanced Tunnel Spin Injection into Graphene Using Chemical Vapor Deposited Hexagonal Boron Nitride. Sci. Rep 2014, 4, 6146 10.1038/srep06146. 25156685 PMC4143790

[ref46] Britnell L.; Gorbachev R. V.; Jalil R.; Belle B. D.; Schedin F.; Katsnelson M. I.; Eaves L.; Morozov S. V.; Mayorov A. S.; Peres N. M. R.; Castro Neto A. H.; Leist J.; Geim A. K.; Ponomarenko L. A.; Novoselov K. S. Electron Tunneling through Ultrathin Boron Nitride Crystalline Barriers. Nano Lett. 2012, 12 (3), 1707–1710. 10.1021/nl3002205. 22380756

[ref47] Maity A.; Grenadier S. J.; Li J.; Lin J. Y.; Jiang H. X. Hexagonal Boron Nitride Neutron Detectors with High Detection Efficiencies. J. Appl. Phys. 2018, 123 (4), 44501 10.1063/1.5017979.

[ref48] Doan T. C.; Li J.; Lin J. Y.; Jiang H. X. Growth and Device Processing of Hexagonal Boron Nitride Epilayers for Thermal Neutron and Deep Ultraviolet Detectors. AIP Adv. 2016, 6 (7), 75213 10.1063/1.4959595.

[ref49] Doan T. C.; Majety S.; Grenadier S.; Li J.; Lin J. Y.; Jiang H. X. Fabrication and Characterization of Solid-State Thermal Neutron Detectors Based on Hexagonal Boron Nitride Epilayers. Nucl. Instrum Methods Phys. Res. A 2014, 748, 84–90. 10.1016/j.nima.2014.02.031.

[ref50] Maity A.; Doan T. C.; Li J.; Lin J. Y.; Jiang H. X. Realization of Highly Efficient Hexagonal Boron Nitride Neutron Detectors. Appl. Phys. Lett. 2016, 109 (7), 72101 10.1063/1.4960522.

[ref51] Doan T. C.; Majety S.; Grenadier S.; Li J.; Lin J. Y.; Jiang H. X. Hexagonal Boron Nitride Thin Film Thermal Neutron Detectors with High Energy Resolution of the Reaction Products. Nucl. Instrum Methods Phys. Res. A 2015, 783, 121–127. 10.1016/j.nima.2015.02.045.

[ref52] Maity A.; Grenadier S. J.; Li J.; Lin J. Y.; Jiang H. X. Toward Achieving Flexible and High Sensitivity Hexagonal Boron Nitride Neutron Detectors. Appl. Phys. Lett. 2017, 111 (3), 33507 10.1063/1.4995399.

[ref53] Li J.; Dahal R.; Majety S.; Lin J. Y.; Jiang H. X. Hexagonal Boron Nitride Epitaxial Layers as Neutron Detector Materials. Nucl. Instrum Methods Phys. Res. A 2011, 654 (1), 417–420. 10.1016/j.nima.2011.07.040.

[ref54] Watanabe K.; Taniguchi T.; Kanda H. Direct-Bandgap Properties and Evidence for Ultraviolet Lasing of Hexagonal Boron Nitride Single Crystal. Nat. Mater. 2004, 3 (6), 404–409. 10.1038/nmat1134. 15156198

[ref55] Watanabe K.; Taniguchi T.; Niiyama T.; Miya K.; Taniguchi M. Far-Ultraviolet Plane-Emission Handheld Device Based on Hexagonal Boron Nitride. Nat. Photonics 2009, 3, 591 10.1038/nphoton.2009.167.

[ref56] Sun Z.; Martinez A.; Wang F. Optical Modulators with 2D Layered Materials. Nat. Photonics 2016, 10, 227 10.1038/nphoton.2016.15.

[ref57] Caldwell J. D.; Aharonovich I.; Cassabois G.; Edgar J. H.; Gil B.; Basov D. N. Photonics with Hexagonal Boron Nitride. Nat. Rev. Mater. 2019, 4 (8), 552–567. 10.1038/s41578-019-0124-1.

[ref58] Caldwell J. D.; Kretinin A. V.; Chen Y.; Giannini V.; Fogler M. M.; Francescato Y.; Ellis C. T.; Tischler J. G.; Woods C. R.; Giles A. J.; Hong M.; Watanabe K.; Taniguchi T.; Maier S. A.; Novoselov K. S. Sub-Diffractional Volume-Confined Polaritons in the Natural Hyperbolic Material Hexagonal Boron Nitride. Nat. Commun. 2014, 5, 5221 10.1038/ncomms6221. 25323633

[ref59] Liu Z.; Gong Y.; Zhou W.; Ma L.; Yu J.; Idrobo J. C.; Jung J.; MacDonald A. H.; Vajtai R.; Lou J.; Ajayan P. M. Ultrathin High-Temperature Oxidation-Resistant Coatings of Hexagonal Boron Nitride. Nat. Commun. 2013, 4, 2541 10.1038/ncomms3541. 24092019

[ref60] Kuznia J. N.; Khan M. A.; Olson D. T.; Kaplan R.; Freitas J. Influence of Buffer Layers on the Deposition of High Quality Single Crystal GaN over Sapphire Substrates. J. Appl. Phys. 1993, 73 (9), 4700–4702. 10.1063/1.354069.

[ref61] Kobayashi Y.; Kumakura K.; Akasaka T.; Makimoto T. Layered Boron Nitride as a Release Layer for Mechanical Transfer of GaN-Based Devices. Nature 2012, 484 (7393), 223–227. 10.1038/nature10970. 22498627

[ref62] Kidambi P. R.; Ducati C.; Dlubak B.; Gardiner D.; Weatherup R. S.; Martin M.-B.; Seneor P.; Coles H.; Hofmann S. The Parameter Space of Graphene Chemical Vapor Deposition on Polycrystalline Cu. J. Phys. Chem. C 2012, 116 (42), 22492–22501. 10.1021/jp303597m.

[ref63] Kidambi P. R.; Nguyen G. D.; Zhang S.; Chen Q.; Kong J.; Warner J.; Li A.-P.; Karnik R. Facile Fabrication of Large-Area Atomically Thin Membranes by Direct Synthesis of Graphene with Nanoscale Porosity. Adv. Mater. 2018, 30, 1804977 10.1002/adma.201804977. 30368941

[ref64] Cheng P.; Kelly M. M.; Moehring N. K.; Ko W.; Li A. P.; Idrobo J. C.; Boutilier M. S. H.; Kidambi P. R. Facile Size-Selective Defect Sealing in Large-Area Atomically Thin Graphene Membranes for Sub-Nanometer Scale Separations. Nano Lett. 2020, 20 (8), 5951–5959. 10.1021/acs.nanolett.0c01934. 32628858

[ref65] Cheng P.; Ferrell N.; Hus S. M.; Moehring N. K.; Coupin M. J.; Warner J.; Li A.-P.; Fissell W. H.; Kidambi P. R. Protein-Enabled Size-Selective Defect-Sealing of Atomically Thin 2D Membranes for Dialysis and Nanoscale Separations. Nano Lett. 2025, 25 (1), 193–203. 10.1021/acs.nanolett.4c04706. 39714067 PMC11719630

[ref66] Cheng P.; Fornasiero F.; Jue M. L.; Ko W.; Li A. P.; Idrobo J. C.; Boutilier M. S. H.; Kidambi P. R. Differences in Water and Vapor Transport through Angstrom-Scale Pores in Atomically Thin Membranes. Nat. Commun. 2022, 10.1038/s41467-022-34172-1. PMC9640652 36344569

[ref67] Zhang S.; Wu N.; Zeng Z.; Song R.; Han X.; Chen X.; Hou D.; Yao A.; Wang L. Tunable Ion Transport across Graphene through Tailoring Grain Boundaries. Cell Rep. Phys. Sci. 2022, 3 (7), 100947 10.1016/j.xcrp.2022.100947.

[ref68] Goethem C. V.; Shen Y.; Chi H.-Y.; Mensi M.; Zhao K.; Nijmeijer A.; Just P.-E.; Agrawal K. V. Advancing Molecular Sieving via Å-Scale Pore Tuning in Bottom-Up Graphene Synthesis. ACS Nano 2024, 18 (7), 5730–5740. 10.1021/acsnano.3c11885. 38324377 PMC10883125

[ref69] Kocaman C.; Bondaz L.; Rezaei M.; Hao J.; Agrawal K. V. Direct Synthesis of Nanocrystalline Single-Layer Porous Graphene for Hydrogen Sieving. Carbon 2024, 221, 118866 10.1016/j.carbon.2024.118866.

[ref70] Yuan Z.; He G.; Faucher S.; Kuehne M.; Li S. X.; Blankschtein D.; Strano M. S. Direct Chemical Vapor Deposition Synthesis of Porous Single-Layer Graphene Membranes with High Gas Permeances and Selectivities. Adv. Mater. 2021, 33 (44), 2104308 10.1002/adma.202104308. 34510595

[ref71] Wang J.; Cheng C.; Zheng X.; Idrobo J. C.; Lu A.-Y.; Park J.-H.; Shin B. G.; Jung S. J.; Zhang T.; Wang H.; Gao G.; Shin B.; Jin X.; Ju L.; Han Y.; Li L.-J.; Karnik R.; Kong J. Cascaded Compression of Size Distribution of Nanopores in Monolayer Graphene. Nature 2023, 623 (7989), 956–963. 10.1038/s41586-023-06689-y. 38030784

[ref72] Moehring N. K.; Mansoor Basha A. B.; Chaturvedi P.; Knight T.; Fan X.; Pintauro P. N.; Boutilier M. S. H.; Karan K.; Kidambi P. R. Overcoming the Conductance versus Crossover Trade-off in State-of-the-Art Proton Exchange Fuel-Cell Membranes by Incorporating Atomically Thin Chemical Vapor Deposition Graphene. Nano Lett. 2025, 25, 1165 10.1021/acs.nanolett.4c05725. 39803947 PMC11760178

[ref73] Kidambi P. R.; Mariappan D. D.; Dee N. T.; Vyatskikh A.; Zhang S.; Karnik R.; Hart A. J. A Scalable Route to Nanoporous Large-Area Atomically Thin Graphene Membranes by Roll-to-Roll Chemical Vapor Deposition and Polymer Support Casting. ACS Appl. Mater. Interfaces 2018, 10 (12), 10369–10378. 10.1021/acsami.8b00846. 29553242

[ref74] Cheng P.; Moehring N. K.; Idrobo J. C.; Ivanov I. N.; Kidambi P. R. Scalable Synthesis of Nanoporous Atomically Thin Graphene Membranes for Dialysis and Molecular Separations via Facile Isopropanol-Assisted Hot Lamination. Nanoscale 2021, 13 (5), 2825–2837. 10.1039/D0NR07384A. 33508042

[ref75] Cheng P.; Kelly M. M.; Moehring N. K.; Ko W.; Li A.-P.; Idrobo J. C.; Boutilier M. S. H.; Kidambi P. R. Facile Size-Selective Defect Sealing in Large-Area Atomically Thin Graphene Membranes for Sub-Nanometer Scale Separations. Nano Lett. 2020, 20 (8), 5951–5959. 10.1021/acs.nanolett.0c01934. 32628858

[ref76] Cheng P.; Ferrell N.; Öberg C. M.; Buchsbaum S. F.; Jue M. L.; Park S. J.; Wang D.; Roy S.; Fornasiero F.; Fissell W. H.; Kidambi P. R. High-Performance Hemofiltration via Molecular Sieving and Ultra-Low Friction in Carbon Nanotube Capillary Membranes. Adv. Funct Mater. 2023, 33 (50), 2304672 10.1002/adfm.202304672.

[ref77] Wang D.; Ferrell N. In Vitro Models to Evaluate Molecular Permeability of the Kidney Filtration Barrier BT - Kidney Research: Experimental Protocols; Hewitson T. D., Toussaint N. D., Smith E. R., Eds.; Springer US: New York, NY, 2023; pp 41–53, DOI: 10.1007/978-1-0716-3179-9_4. 37423981

[ref78] Ohlson M.; Sörensson J.; Haraldsson B. Glomerular Size and Charge Selectivity in the Rat as Revealed by FITC-Ficoll and Albumin. American Journal of Physiology-Renal Physiology 2000, 279 (1), F84–F91. 10.1152/ajprenal.2000.279.1.F84. 10894790

[ref79] Kidambi P. R.; Nguyen G. D.; Zhang S.; Chen Q.; Kong J.; Warner J.; Li A.-P.; Karnik R. Facile Fabrication of Large-Area Atomically Thin Membranes by Direct Synthesis of Graphene with Nanoscale Porosity. Adv. Mater. 2018, 30 (49), 1804977 10.1002/adma.201804977. 30368941

[ref80] Kidambi P. R.; Blume R.; Kling J.; Wagner J. B.; Baehtz C.; Weatherup R. S.; Schloegl R.; Bayer B. C.; Hofmann S. In Situ Observations during Chemical Vapor Deposition of Hexagonal Boron Nitride on Polycrystalline Copper. Chem. Mater. 2014, 26 (22), 6380–6392. 10.1021/cm502603n. 25673919 PMC4311958

[ref81] Bae S.; Kim H.; Lee Y.; Xu X.; Park J.-S.; Zheng Y.; Balakrishnan J.; Lei T.; Ri Kim H.; Song Y. Il; Kim Y.-J.; Kim K. S.; Özyilmaz B.; Ahn J.-H.; Hong B. H.; Iijima S. Roll-to-Roll Production of 30-Inch Graphene Films for Transparent Electrodes. Nat. Nanotechnol 2010, 5 (8), 574–578. 10.1038/nnano.2010.132. 20562870

[ref82] Tavakoli M. M.; Azzellino G.; Hempel M.; Lu A.-Y.; Martin-Martinez F. J.; Zhao J.; Yeo J.; Palacios T.; Buehler M. J.; Kong J. Synergistic Roll-to-Roll Transfer and Doping of CVD-Graphene Using Parylene for Ambient-Stable and Ultra-Lightweight Photovoltaics. Adv. Funct Mater. 2020, 30 (31), 2001924 10.1002/adfm.202001924.

[ref83] Juang Z.-Y.; Wu C.-Y.; Lu A.-Y.; Su C.-Y.; Leou K.-C.; Chen F.-R.; Tsai C.-H. Graphene Synthesis by Chemical Vapor Deposition and Transfer by a Roll-to-Roll Process. Carbon 2010, 48 (11), 3169–3174. 10.1016/j.carbon.2010.05.001.

[ref84] Kidambi P. R.; Mariappan D. D.; Dee N. T.; Vyatskikh A.; Zhang S.; Karnik R.; Hart A. J. A Scalable Route to Nanoporous Large-Area Atomically Thin Graphene Membranes by Roll-to-Roll Chemical Vapor Deposition and Polymer Support Casting. ACS Appl. Mater. Interfaces 2018, 10 (12), 10369–10378. 10.1021/acsami.8b00846. 29553242

[ref85] Kidambi P. R.; Terry R. A.; Wang L.; Boutilier M. S. H.; Jang D.; Kong J.; Karnik R. Assessment and Control of the Impermeability of Graphene for Atomically Thin Membranes and Barriers. Nanoscale 2017, 9 (24), 8496–8507. 10.1039/C7NR01921A. 28604878

[ref86] Kidambi P. R.; Boutilier M. S. H.; Wang L.; Jang D.; Kim J.; Karnik R. Selective Nanoscale Mass Transport across Atomically Thin Single Crystalline Graphene Membranes. Adv. Mater. 2017, 29 (19), 1605896 10.1002/adma.201605896. 28306180

[ref87] Babenko V.; Lane G.; Koos A. A.; Murdock A. T.; So K.; Britton J.; Meysami S. S.; Moffat J.; Grobert N. Time Dependent Decomposition of Ammonia Borane for the Controlled Production of 2D Hexagonal Boron Nitride. Sci. Rep 2017, 7 (1), 14297 10.1038/s41598-017-14663-8. 29085080 PMC5662770

[ref88] Song X.; Li Q.; Ji J.; Yan Z.; Gu Y.; Huo C.; Zou Y.; Zhi C.; Zeng H. A Comprehensive Investigation on CVD Growth Thermokinetics of H-BN White Graphene. 2d Mater. 2016, 3 (3), 035007 10.1088/2053-1583/3/3/035007.

[ref89] Makarova A. A.; Fernandez L.; Usachov D. Yu.; Fedorov A.; Bokai K. A.; Smirnov D. A.; Laubschat C.; Vyalikh D. V.; Schiller F.; Ortega J. E. Oxygen Intercalation and Oxidation of Atomically Thin H-BN Grown on a Curved Ni Crystal. J. Phys. Chem. C 2019, 123 (1), 593–602. 10.1021/acs.jpcc.8b10574.

[ref90] Babenko V.; Fan Y.; Veigang-Radulescu V.-P.; Brennan B.; Pollard A. J.; Burton O.; Alexander-Webber J. A.; Weatherup R. S.; Canto B.; Otto M.; Neumaier D.; Hofmann S. Oxidising and Carburising Catalyst Conditioning for the Controlled Growth and Transfer of Large Crystal Monolayer Hexagonal Boron Nitride. 2d Mater. 2020, 7 (2), 024005 10.1088/2053-1583/ab6269.

[ref91] Zhang J.; Yang Y.; Lou J. Investigation of Hexagonal Boron Nitride as an Atomically Thin Corrosion Passivation Coating in Aqueous Solution. Nanotechnology 2016, 27 (36), 364004 10.1088/0957-4484/27/36/364004. 27483462

[ref92] Li X.; Long Y.; Ma L.; Li J.; Yin J.; Guo W. Coating Performance of Hexagonal Boron Nitride and Graphene Layers. 2d Mater. 2021, 8 (3), 034002 10.1088/2053-1583/abe777.

[ref93] Kim K. K.; Hsu A.; Jia X.; Kim S. M.; Shi Y.; Hofmann M.; Nezich D.; Rodriguez-Nieva J. F.; Dresselhaus M.; Palacios T.; Kong J. Synthesis of Monolayer Hexagonal Boron Nitride on Cu Foil Using Chemical Vapor Deposition. Nano Lett. 2012, 12 (1), 161–166. 10.1021/nl203249a. 22111957

[ref94] Guo N.; Wei J.; Fan L.; Jia Y.; Liang D.; Zhu H.; Wang K.; Wu D. Controllable Growth of Triangular Hexagonal Boron Nitride Domains on Copper Foils by an Improved Low-Pressure Chemical Vapor Deposition Method. Nanotechnology 2012, 23 (41), 415605 10.1088/0957-4484/23/41/415605. 23011199

[ref95] Wu Q.; Park J.-H.; Park S.; Jung S. J.; Suh H.; Park N.; Wongwiriyapan W.; Lee S.; Lee Y. H.; Song Y. J. Single Crystalline Film of Hexagonal Boron Nitride Atomic Monolayer by Controlling Nucleation Seeds and Domains. Sci. Rep 2015, 5 (1), 16159 10.1038/srep16159. 26537788 PMC4633619

[ref96] Song X.; Gao J.; Nie Y.; Gao T.; Sun J.; Ma D.; Li Q.; Chen Y.; Jin C.; Bachmatiuk A.; Rümmeli M. H.; Ding F.; Zhang Y.; Liu Z. Chemical Vapor Deposition Growth of Large-Scale Hexagonal Boron Nitride with Controllable Orientation. Nano Res. 2015, 8 (10), 3164–3176. 10.1007/s12274-015-0816-9.

[ref97] Stehle Y.; Meyer H. M.; Unocic R. R.; Kidder M.; Polizos G.; Datskos P. G.; Jackson R.; Smirnov S. N.; Vlassiouk I. V. Synthesis of Hexagonal Boron Nitride Monolayer: Control of Nucleation and Crystal Morphology. Chem. Mater. 2015, 27 (23), 8041–8047. 10.1021/acs.chemmater.5b03607.

[ref98] Kidambi P. R.; Ducati C.; Dlubak B.; Gardiner D.; Weatherup R. S.; Martin M.-B.; Seneor P.; Coles H.; Hofmann S. The Parameter Space of Graphene Chemical Vapor Deposition on Polycrystalline Cu. J. Phys. Chem. C 2012, 116 (42), 22492–22501. 10.1021/jp303597m.

[ref99] Diulus J. T.; Naclerio A. E.; Boscoboinik J. A.; Head A. R.; Strelcov E.; Kidambi P. R.; Kolmakov A. Operando XPS for Plasma Process Monitoring: A Case Study on the Hydrogenation of Copper Oxide Confined under h-BN. J. Phys. Chem. C 2024, 128 (18), 7591–7600. 10.1021/acs.jpcc.4c00253.

[ref100] Lin W.-H.; Brar V. W.; Jariwala D.; Sherrott M. C.; Tseng W.-S.; Wu C.-I.; Yeh N.-C.; Atwater H. A. Atomic-Scale Structural and Chemical Characterization of Hexagonal Boron Nitride Layers Synthesized at the Wafer-Scale with Monolayer Thickness Control. Chem. Mater. 2017, 29 (11), 4700–4707. 10.1021/acs.chemmater.7b00183.

[ref101] Reich S.; Ferrari A. C.; Arenal R.; Loiseau A.; Bello I.; Robertson J. Resonant Raman Scattering in Cubic and Hexagonal Boron Nitride. Phys. Rev. B 2005, 71 (20), 205201 10.1103/PhysRevB.71.205201.

[ref102] Shi Y.; Hamsen C.; Jia X.; Kim K. K.; Reina A.; Hofmann M.; Hsu A. L.; Zhang K.; Li H.; Juang Z.-Y.; Dresselhaus Mildred. S.; Li L.-J.; Kong J. Synthesis of Few-Layer Hexagonal Boron Nitride Thin Film by Chemical Vapor Deposition. Nano Lett. 2010, 10 (10), 4134–4139. 10.1021/nl1023707. 20812716

[ref103] Kim G.; Jang A.-R.; Jeong H. Y.; Lee Z.; Kang D. J.; Shin H. S. Growth of High-Crystalline, Single-Layer Hexagonal Boron Nitride on Recyclable Platinum Foil. Nano Lett. 2013, 13 (4), 1834–1839. 10.1021/nl400559s. 23527543

[ref104] Gao Y.; Ren W.; Ma T.; Liu Z.; Zhang Y.; Liu W.-B.; Ma L.-P.; Ma X.; Cheng H.-M. Repeated and Controlled Growth of Monolayer, Bilayer and Few-Layer Hexagonal Boron Nitride on Pt Foils. ACS Nano 2013, 7 (6), 5199–5206. 10.1021/nn4009356. 23663007

[ref105] Elias C.; Valvin P.; Pelini T.; Summerfield A.; Mellor C. J.; Cheng T. S.; Eaves L.; Foxon C. T.; Beton P. H.; Novikov S. V.; Gil B.; Cassabois G. Direct Band-Gap Crossover in Epitaxial Monolayer Boron Nitride. Nat. Commun. 2019, 10 (1), 2639 10.1038/s41467-019-10610-5. 31201328 PMC6572751

[ref106] Lobo V. M. M.; Ribeiro A. C. F.; Verissimo L. M. P. Diffusion Coefficients in Aqueous Solutions of Potassium Chloride at High and Low Concentrations. J. Mol. Liq. 1998, 78 (1), 139–149. 10.1016/S0167-7322(98)00088-9.

[ref107] Vitagliano V.; Lyons P. A. Diffusion Coefficients for Aqueous Solutions of Sodium Chloride and Barium Chloride. J. Am. Chem. Soc. 1956, 78 (8), 1549–1552. 10.1021/ja01589a011.

[ref108] Lapidus L. J.; Eaton W. A.; Hofrichter J. Measuring the Rate of Intramolecular Contact Formation in Polypeptides. Proc. Natl. Acad. Sci. U. S. A. 2000, 97 (13), 7220–7225. 10.1073/pnas.97.13.7220. 10860987 PMC16526

[ref109] Dembczynski R.; Jankowski T. Characterisation of Small Molecules Diffusion in Hydrogel-Membrane Liquid-Core Capsules. Biochem Eng. J. 2000, 6 (1), 41–44. 10.1016/S1369-703X(00)00070-X. 10908867

[ref110] Kim Y.-C.; Myerson A. S. Diffusivity of Lysozyme in Undersaturated, Saturated and Supersaturated Solutions. J. Cryst. Growth 1994, 143 (1), 79–85. 10.1016/0022-0248(94)90370-0.

[ref111] Cheng P.; Fornasiero F.; Jue M. L.; Ko W.; Li A.-P.; Idrobo J. C.; Boutilier M. S. H.; Kidambi P. R. Differences in Water and Vapor Transport through Angstrom-Scale Pores in Atomically Thin Membranes. Nat. Commun. 2022, 13 (1), 6709 10.1038/s41467-022-34172-1. 36344569 PMC9640652

[ref112] Frueh S.; Kellett R.; Mallery C.; Molter T.; Willis W. S.; King’ondu C.; Suib S. L. Pyrolytic Decomposition of Ammonia Borane to Boron Nitride. Inorg. Chem. 2011, 50 (3), 783–792. 10.1021/ic101020k. 21182274

[ref113] Wood G. E.; Laker Z. P. L.; Marsden A. J.; Bell G. R.; Wilson N. R. In Situ Gas Analysis during the Growth of Hexagonal Boron Nitride from Ammonia Borane. Mater. Res. Express 2017, 4 (11), 115905 10.1088/2053-1591/aa9a7f.

[ref114] Sharma S.; Kalita G.; Vishwakarma R.; Zulkifli Z.; Tanemura M. Opening of Triangular Hole in Triangular-Shaped Chemical Vapor Deposited Hexagonal Boron Nitride Crystal. Sci. Rep 2015, 5 (1), 10426 10.1038/srep10426. 25994455 PMC4650756

