# Supplementary material for: Scalable Bottom-Up Synthesis of Nanoporous Hexagonal Boron Nitride (h-BN) for Large-Area Atomically Thin Ceramic Membranes
Source: Nano Lett. 2025 Feb 14;25(8):3221–32. doi: 10.1021/acs.nanolett.4c05939 (PMC11869279; doi:10.1021/acs.nanolett.4c05939)
Supplement: Supplementary file 1 — nl4c05939_si_001.pdf [file nl4c05939_si_001.pdf]

Supporting information:

Scalable Bottom-Up Synthesis of Nanoporous Hexagonal Boron Nitride (h-BN) for Large-Area Atomically Thin Ceramic Membranes

Andrew E. Naclerio[1⊥], *Peifu Cheng*[1⊥], *Saban M. Hus*[2], *J. Trey Diulus*[3], *Marti Checa*[2], *Ivan Vlassiouk*[2], *William H. Fissell*[4,5], *Matthew Coupin,*[7] *Jamie Warner,*[7] *Liam Collins*[2], *Andrei Kolmakov*[3], *An-Ping Li*[2], *Piran R. Kidambi*[1,5,6,7,*]

[1]Department of Chemical and Biomolecular Engineering, Vanderbilt University, Nashville, TN 37212, USA
[2]Center for Nanophase Materials Sciences, Oak Ridge National Laboratory, Oak Ridge, TN 37831, USA
[3]Nanoscale Device characterization Division, PML, NIST, Gaithersberg, MD 20899, USA
[4]Department of Medicine and Division of Nephrology and Hypertension, Vanderbilt University Medical Center, Nashville, TN 37232, USA
[5]Vanderbilt Institute of Nanoscale Sciences and Engineering, Vanderbilt University, Nashville, TN 37212, USA
[6]Department of Mechanical Engineering, Vanderbilt University, Nashville, TN 37212, USA
[7]Walker Department of Mechanical Engineering, University of Texas at Austin, Austin, TX 78712, USA.

[⊥]These authors contributed equally to this publication.
[*]Email: piran.kidambi@vanderbilt.edu

Experimental methods

h-BN Synthesis

h-BN is grown via low-pressure chemical vapor deposition (LPCVD) on polycrystalline Cu foil (99.9% purity, 18 µm thick, JX Holding) in a hot walled tube furnace reactor using H_2 gas and ammonia-borane (H_3BNH_3) precursor (~3-3.5 mg, 99%, Sigma Aldrich).[1,2] The precursor is fed into the reactor in vapor form by heating it in a separate chamber upstream from the furnace (Figure 1A).[3–5] A thermocouple and temperature controller allowed controlled heating of the side chamber and gas line leading into the system (Figure S6). A ball valve allows isolation of the precursor chamber from the reaction zone.[5]

The Cu foil catalyst is initially rinsed in acetone and IPA to remove organic residues, and then sonicated in 20% nitric acid for 3 min to remove surface oxides or contaminants, followed by de-ionized (DI) water rinsing and dried in air before loading into the LPCVD reactor.[6,7] Next, the foil is heated in ~500 sccm of H_2 to ~1025 ℃ at 40 ℃/min and annealed for 30 minutes. After annealing, the furnace temperature is reduced to the desired growth temperature (875 ℃ - 1025 ℃ ±15 ℃), H_2 flow rate is set to ~50sccm and the ammonia-borane precursor introduced by setting the desired side chamber temperature (~60-90 ℃) to synthesize *h*-BN (Figure 1B). Typical growth pressure is ~500 mTorr. After the desired growth time, the precursor delivery is turned off and the reactor is quench cooled[8–10]. Unless stated otherwise, *h*-BN was grown using ~3.5 mg of ammonia-borane heated at 85 ℃ for 90 minutes. AFM, Raman, and absorption measurements were performed on *h*-BN grown at 1025℃.

h-BN Transfer

h-BN is transferred to 300nm SiO_2/Si wafer and fused silica substrates using a poly(methyl methacrylate) (PMMA) carrier layer.[9–13] Briefly, PMMA (~4 wt% in anisole) is spin-coated (1500 rpm for 60 seconds) onto the *h*-BN on Cu foil, heated on a hot plate to 90 ℃ before etching the Cu foil in an ammonium persulfate solution (APS, 0.2 M). The resulting PMMA/h-BN stack is rinsed with DI water and transferred onto to the desired substrate and heated (90 ℃, 30 min) to ensure uniform contract by removing interfacial water. Finally, the PMMA is dissolved in acetone overnight and rinsed in IPA.

h-BN Transfer to TEM grids

A polymer free process was used to transfer monolayer h-BN to Holey Carbon on 300 Mesh Au grids (Electron Microscopy Sciences) using methods described previously.[14–22] A drop of isopropyl alcohol is added to h-BN on Cu foil placed on the TEM grid (after the h-BN on the other side is removed by etching in 0.2M APS solution). The TEM grid|h-BN|Cu foil stack was floated on 0.2M APS to completely remove the Cu foil via etching, floated on DI water, and rinsing in ethanol and dried in air.

h-BN Characterization

Oxidation of *h*-BN/Cu is performed on a hot plate at ~200 ℃ for ~1-5 minutes to observe the Cu color change. Scanning electron microscopy (SEM) was performed using a Zeiss Merlin Scanning Electron Microscope with Gemini II Column at ~2-5 kV. Image analysis is performed using imageJ.

AFM images were obtained with a Cypher AFM system (from Asylum Research, an Oxford Instruments company). Images were captured in AC mode, using commercially available Multi75-G (BudgetSensors) tips with a free resonance frequency of 75 kHz and a spring constant of 3 N m^{-1}. Setpoint used during imaging was 70% of the free amplitude.

UV-vis absorption spectra were collected on *h*-BN transferred to fused silica on a Cary 60 UV-vis spectrometer using bare fused silica as a baseline. A tauc plot is computed assuming a direct bandgap for monolayer *h*-BN. [5,23–30]

XPS measurements were performed at NIST as described elsewhere.[2] Spectra were collected using a monochromatized Al Kα X-ray source (hν = 1,486.6 eV) with a 1.5 mm × 2 mm spot size angled 30° from normal to the sample surface. The electron analyzer is positioned 90° relative to the X-ray source, resulting in a 30° take-off angle relative to the sample plane.

STM measurements were obtained with an Omicron variable temperature scanning tunneling microscope (VT-STM) at room temperature in the Center for Nanophase Materials Sciences at Oak Ridge National Laboratory. The samples were annealed under ultra-high vacuum at 400 °C for ~5 h before imaging.

Raman spectroscopy is performed on *h*-BN transferred to 300nm SiO_2/Si wafer with a Renishaw Raman spectrometer using 532nm excitation wavelength at Oak Ridge National Laboratory. [23,31,32]

A JEOL ARM200F NEOARM equipped with a CEOS ASCOR corrector operated at 80 kV was used to image monolayer h-BN.[22] ADF-STEM images were acquired with a 40 micron aperature to obtain a convergence semiangle of 27 milliradians for the electron probe. To minimize contaminations prior to imaging the h-BN samples were annealed in vacuum ~175-200°C for ~12 hours.

Transfer to PES support

PES support casting was performed as detailed elsewhere.[33,34] As synthesized *h*-BN on Cu foil is secured on Al plate using Scotch tape on all four edges. Additional 2 layers of Scotch tape are added to two opposite sides of the foil to define the thickness of the casting layers (~150 μm). PES solution (~16 wt% PES + ~ 82 wt% NMP + 2 wt% IPA, baked at 75℃ overnight, cooled, and degassed under vacuum overnight) is casted with a disposable glass culture tube, evenly spreading

an ~150 μm layer of PES solution over the Cu. (see Figure 3A) The sample is then immediately submerged in DI water for 30 minutes to induce phase separation.[33,34] The PES/*h*-BN/Cu is then removed from the Al plate and air dried. Finally, the Cu is etched in 0.2M ammonium persulfate, rinsed in DI water and ethanol, and dried in air to obtain a *h*-BN/PES membrane.

Transport measurements

Transport measurements are performed in a custom side-bi-side glass diffusion cell (Permegear) consisting of ~7 mL reservoirs and a ~5 mm membrane orifice and a gas-tight syringe on the feed side of the cell to induce a hydrostatic head for pressure-driven flow measurements. [9–11,13,34–37] The *h*-BN PES membranes are mounted into the cell and rinsed three times in ethanol in order to enable pore wetting and pressure driven transport of ethanol is used to quantify the coverage of h-BN on PES supports. For an effective PES control membrane, we casted PES on CVD graphene on Cu foil and removed the graphene via O_2 plasma etch (Harrick Plasma PDC-001, 500 mTorr, 300 s, low power).[11] The structure of PES before and after graphene removal was found to be similar from SEM imaging. PES casted on bare Cu did not exhibit a porous morphology similar to PES under h-BN and due to the inert nature of *h*-BN it could not be removed via O_2 plasma necessitating the development of an effective control PES membrane as detailed above.

For diffusive transport of KCl and NaCl, ~7 mL of 0.5 M of salt solution is added to the feed side of the membrane, while DI water is added to the permeate side. [9–11,13,34–37] The change in solution conductivity of the permeate side is measured via a Mettler Toledo SevenCompact S230 conductivity meter every 15 s for 15 min and used to quantify transport. [9–11,13,34–37] For L-Tr, B12, and Lz, 1mM of analyte in 0.5 M KCl solution is introduced on the feed side, while 0.5 M KCl is introduced on the permeate side. [9–11,13,34–37] A fiber optic dip probe connected to an Agilent Cary 60 UV-vis spectrophotometer is placed in the permeate side of the membrane, and the absorbance of the permeate solution from 190 – 1100 nm is collected every 15 s for 40 min. The intensity difference between ~710nm (water) and ~279 nm (L-Tr), ~360 nm (B12), and ~282 nm (Lz) over time is used to computer transport of L-Tr, B12, and Lz, respectively. [9–11,13,34–37]

Permeance is calculated as $P = (V*dC/dt)/(A*\Delta C)$ where V is the volume of the reservoir (7 mL), dC/dt is the slope of permeate side concentration as a function of time, A is the area of the membrane, and ΔC is the concentration difference between the feed and permeate sides. [9–11,13,34–37]

Ficoll Measurements

Ficoll 70 was labelled via conjugation with fluorescein isothiocynate (FITC) and solubilized in PBS solution (0.1 mg mL^{-1}, ~7.4 pH) as described elsewhere. [35,38,39] Ficoll solution is added to the feed side of the cell, and PBS solution is added to the permeate side. Feed and permeate solution are collected after 1 day, 3 days, 5 days, and 7 days. The concentration of Ficoll as a function of size is calculated by size-exclusion chromatography as described elsewhere. [35,38,39]

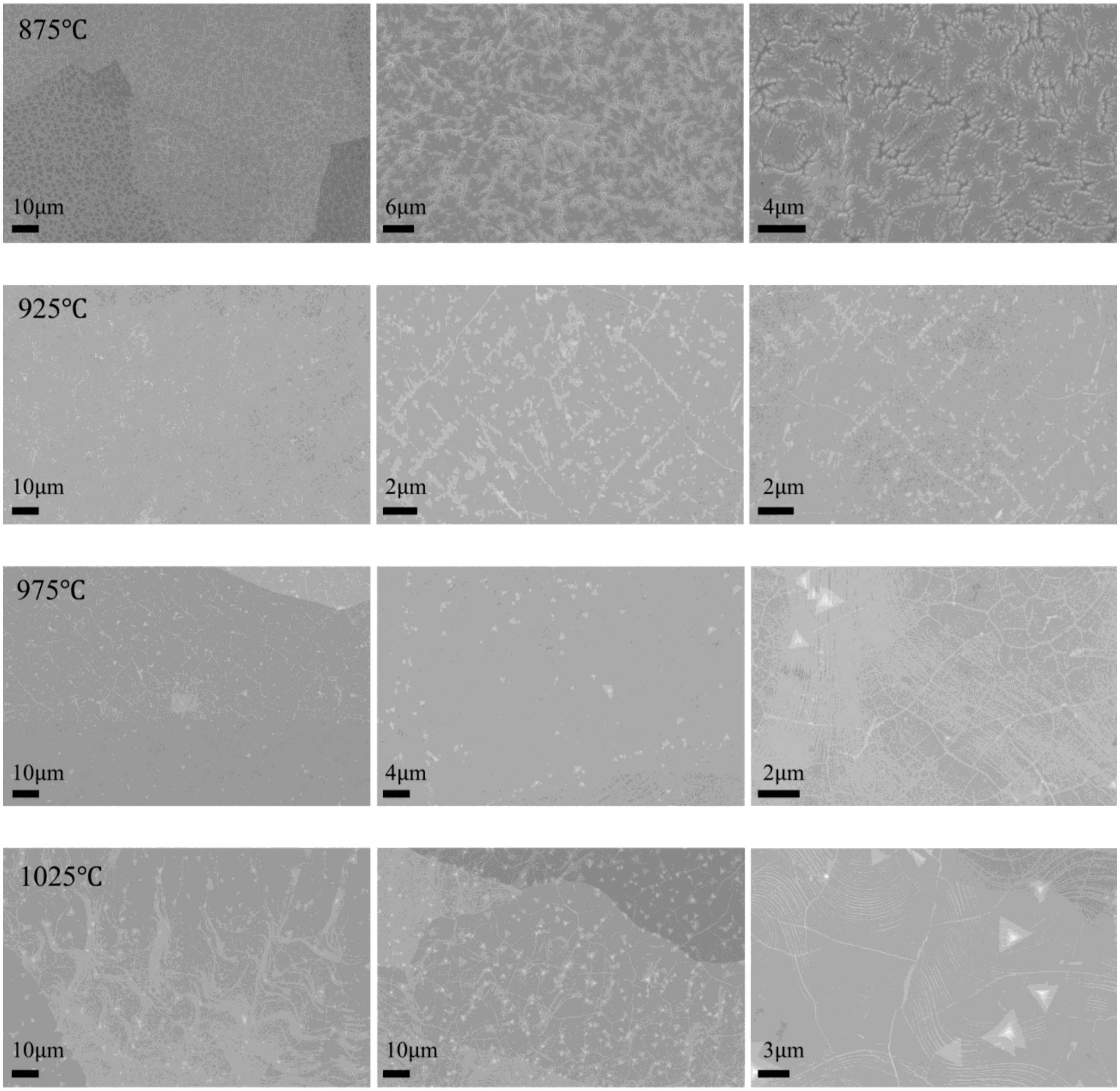

Figure S1. SEM images for *h*-BN synthesized at different temperatures. All samples were grown for ~90 minutes in H_2 ~50 sccm, using ~3.5 mg of precursor heated at ~85 ℃. SEM images (left to right – increasing magnification) showing the surface morphology of *h*-BN on Cu foil synthesized via CVD. *h*-BN grown at 875 °C is observed to be incomplete, hence only *h*-BN grown at ≥925 °C were used for membrane fabrication (Figure 3). A clear change in *h*-BN morphology is observed upon decreasing the growth temperature below 975 °C consistent with Figure 4.

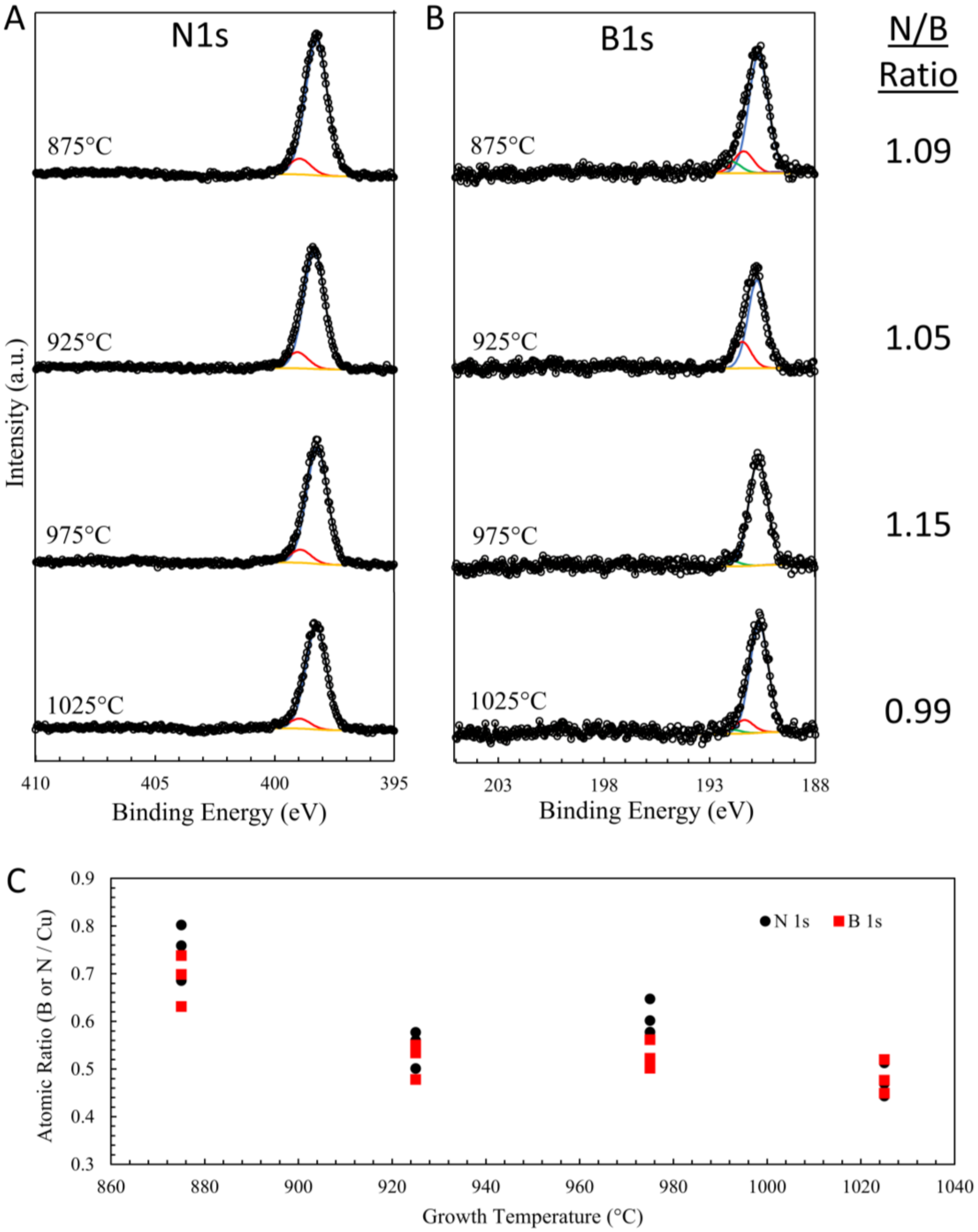

Figure S2. X-ray photoelectron spectroscopy (XPS) analysis for h-BN synthesized at different temperatures. A) N 1s and B) B 1s core level XPS spectra of *h*-BN as-grown on Cu foil at 875 – 1025 ℃ via CVD. Numbers on the right show the computed atomic N:B ratio. Peak fitting are based on prior reports in literature.[2,8,40] C) Atomic ratio of N and B compared to Cu as a function of CVD synthesis temperature. Each ratio is computed for peak area of N 1s or B 1s to Cu 2p, Cu 3s, and Cu 3p, accounting for suitable sensitivity factors.[2,41–43] The absence of significant differences in the XPS spectra or ratios indicates the stoichiometric ratio of B:N ~1 in maintained in the films synthesized at different temperatures despite the change in film morphology (see Figure 4 and Figure S1).

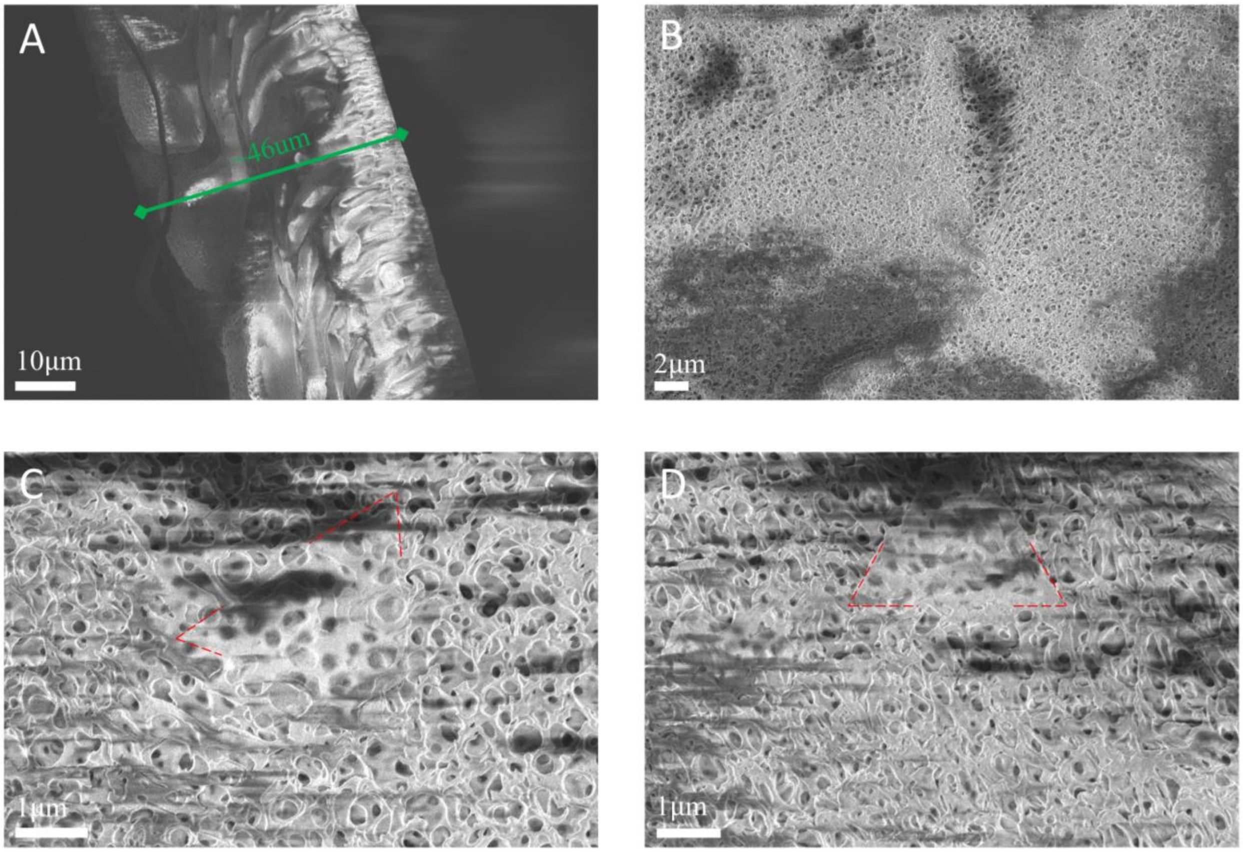

Figure S3. SEM images of *h*-BN film with PES support casting. A) Cross-section SEM image of *h*-BN+ PES membranes indicate the porous PES support is ~46 μm thick. B) Top view SEM image of the hBN supported on PES (lower magnification and wider field of view of Figure 3C). C-D) SEM images of *h*-BN bilayers observed on PES. Dotted red lines are a guide for the eye. The insulating nature of both *h*-BN and PES making imaging suspended monolayer *h*-BN non-trivial. However, the presence of triangular features consistent with the size and shape of bilayers observed under SEM of *h*-BN/Cu (Figure 1 and 2) indicates successful *h*-BN transfer to PES.

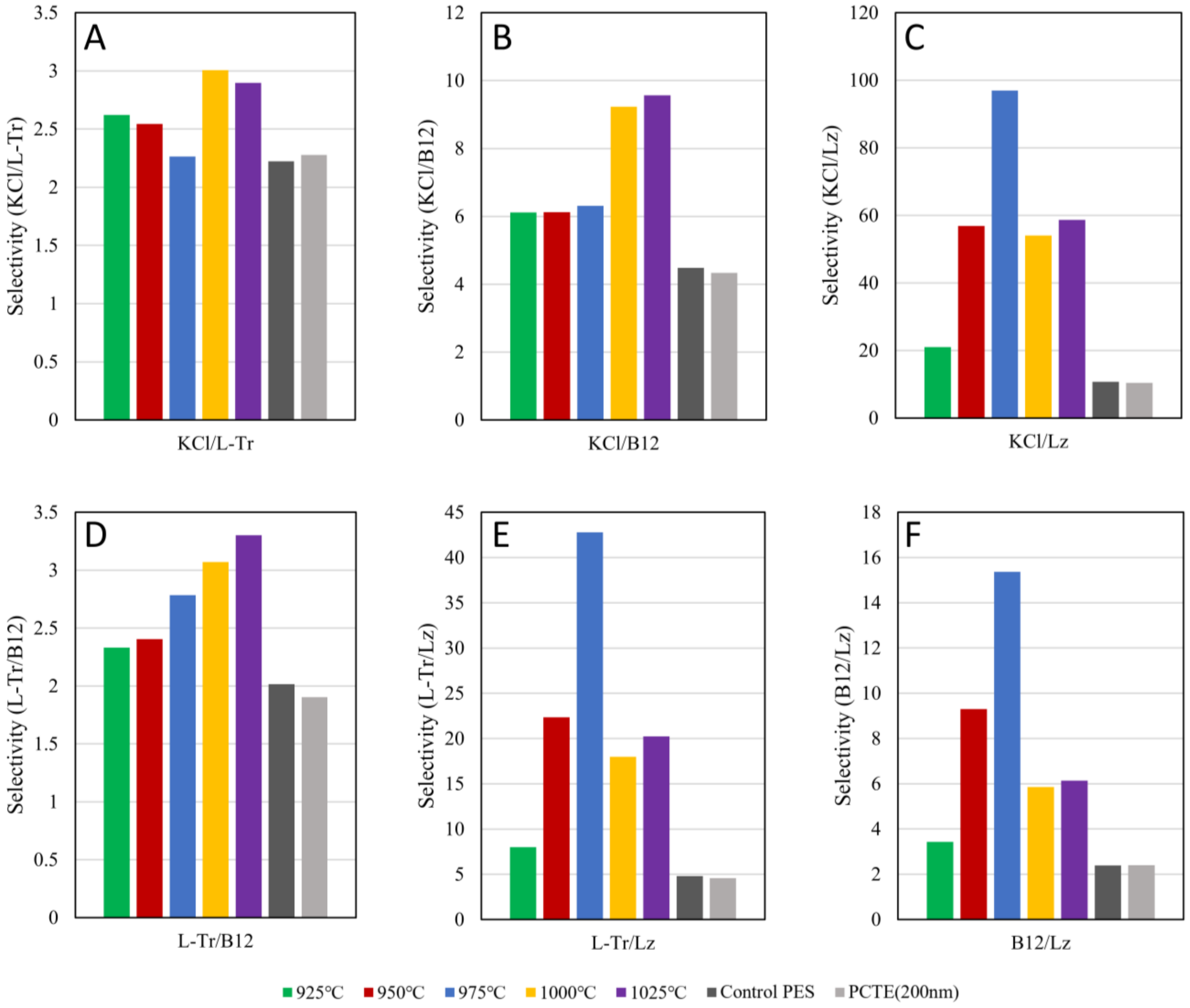

Figure S4. Selectivity of *h*BN+PES membranes.

A) KCl/L-Tr, B) KCl/B12, C) KCl/Lz, D) L-Tr/B12, E) L-Tr/Lz, and F) B12/Lz selectivity computed by taking the ratio of respective permeance values measured for the *h*BN+PES membranes fabricated using *h*-BN synthesized at different temperatures. Also shown are PES membranes (PES casted on CVD graphene on Cu, Cu etched in acid and graphene etched via O_2 plasma) and 200 nm polycarbonate track-etched membranes (Sterlitech, 10 μm thickness, ~10% porosity) as experimental controls and note both PES and PCTE controls show similar selectivity.

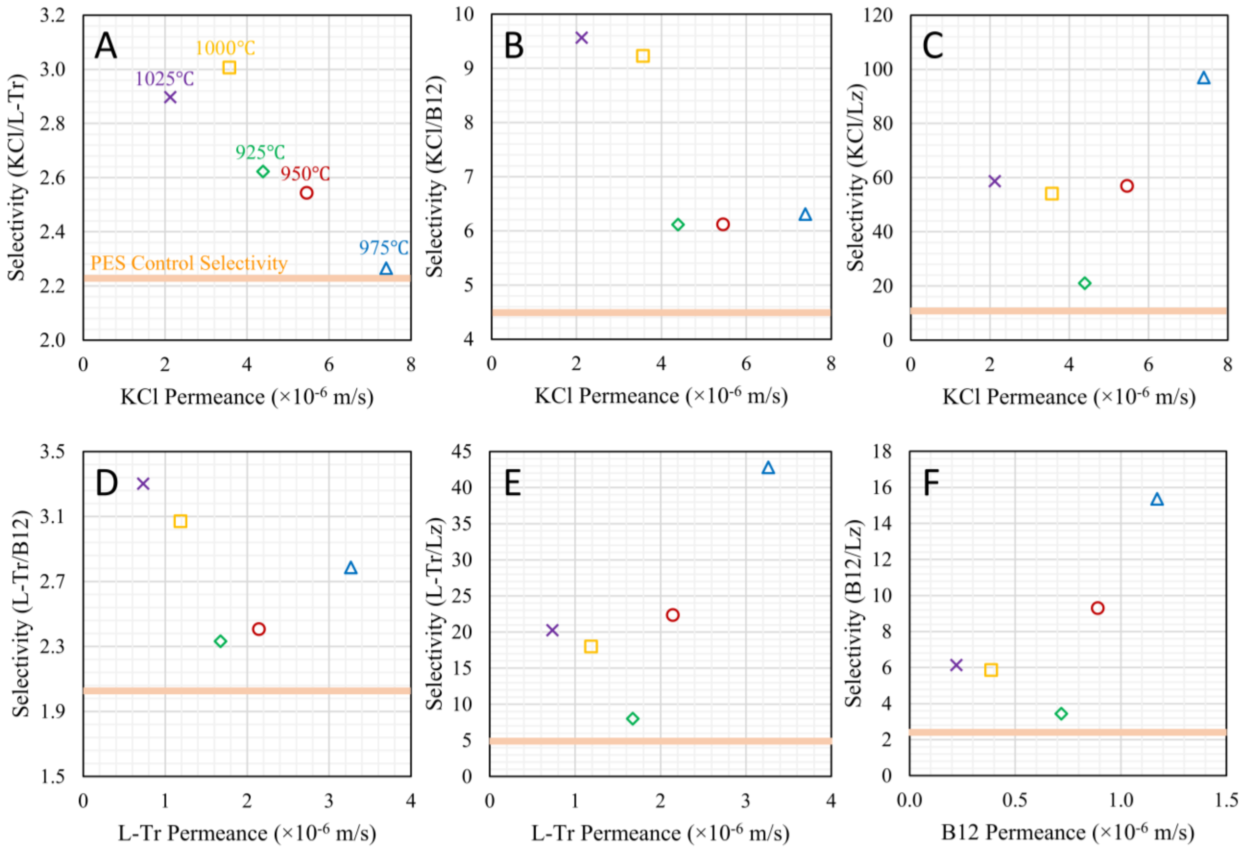

Figure S5. Selectivity as function of permeance for *h*BN+PES membranes.

A) KCl/L-Tr selectivity vs KCl permeance, B) KCl/B12 selectivity vs KCl permeance, C) KCl/Lz selectivity vs KCl permeance, D) L-Tr/B12 selectivity vs L-Tr permeance, E) L-Tr/Lz selectivity vs L-Tr permeance, and F) B12/Lz selectivity vs B12 permeance for the *h*BN+PES membranes fabricated using *h*-BN synthesized at different temperatures. Also shown are PES control membranes (PES casted on CVD graphene on Cu, Cu etched in acid and graphene etched via O_2 plasma) and 200 nm polycarbonate track-etched membranes (Sterlitech, 10 um thickness, ~10% porosity) as experimental controls.

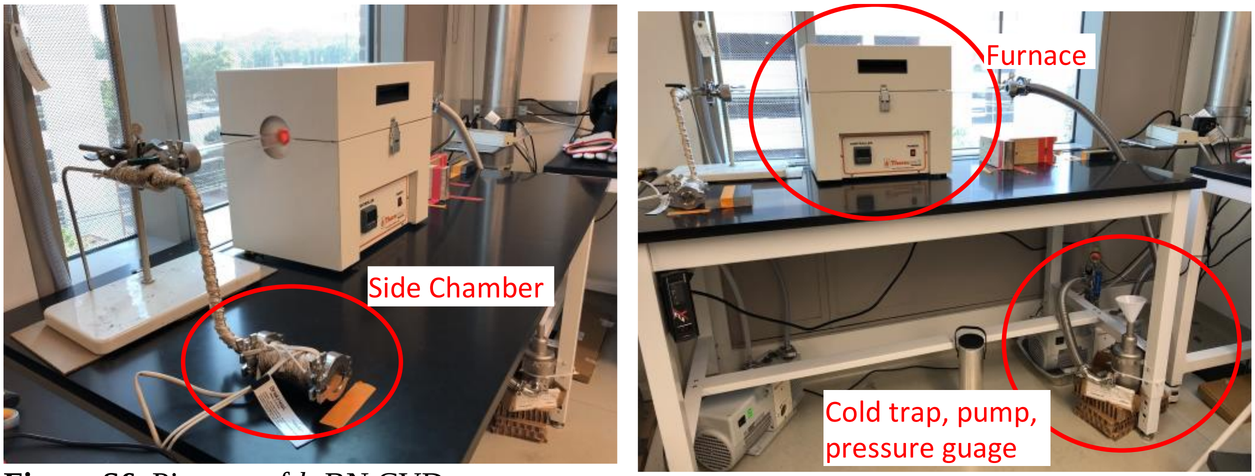

Figure S6. Pictures of *h*-BN CVD system.

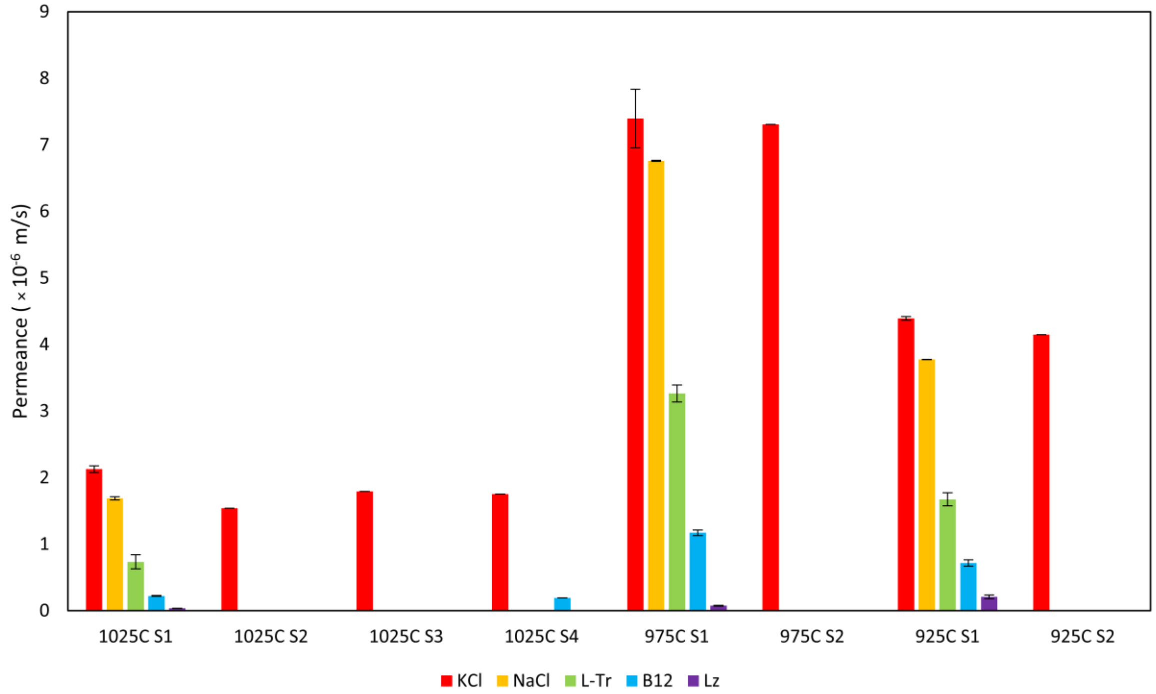

Figure S7. Different *h*-BN membranes on PES supports.
Sample S1 and S2 correspond to different membranes fabricated using identical CVD growths and PES casting processes indicating reproducibility.

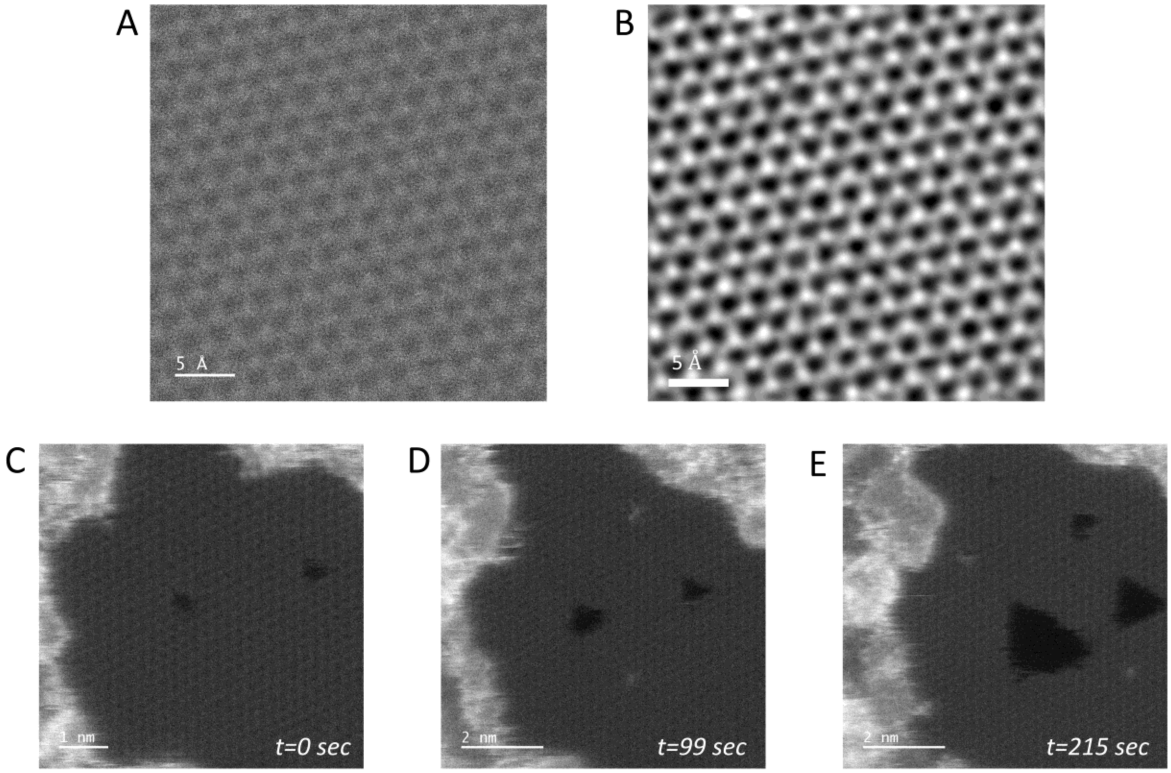

Figure S8. STEM images of *h*-BN grown at 1025°C. A) Raw STEM image acquired at 80kV acceleration voltage. B) Filtered image of A using a low bandpass filter. C-E) STEM images of electron beam induced damage to *h*-BN at 80kV. Triangular pores form and increase in size with continued exposure to the electron beam.

Supporting Information References

(1) Naclerio, A. E.; Kidambi, P. R. A Review of Scalable Hexagonal Boron Nitride (H-BN) Synthesis for Present and Future Applications. *Advanced Materials* **2023**, *35* (6), 2207374.

(2) Diulus, J. T.; Naclerio, A. E.; Boscoboinik, J. A.; Head, A. R.; Strelcov, E.; Kidambi, P. R.; Kolmakov, A. Operando XPS for Plasma Process Monitoring: A Case Study on the Hydrogenation of Copper Oxide Confined under h-BN. *The Journal of Physical Chemistry C* **2024**, *128* (18), 7591–7600. https://doi.org/10.1021/acs.jpcc.4c00253.

(3) Babenko, V.; Lane, G.; Koos, A. A.; Murdock, A. T.; So, K.; Britton, J.; Meysami, S. S.; Moffat, J.; Grobert, N. Time Dependent Decomposition of Ammonia Borane for the Controlled Production of 2D Hexagonal Boron Nitride. *Sci Rep* **2017**, *7* (1), 14297. https://doi.org/10.1038/s41598-017-14663-8.

(4) Wood, G. E.; Laker, Z. P. L.; Marsden, A. J.; Bell, G. R.; Wilson, N. R. In Situ Gas Analysis during the Growth of Hexagonal Boron Nitride from Ammonia Borane. *Mater Res Express* **2017**, *4* (11), 115905. https://doi.org/10.1088/2053-1591/aa9a7f.

(5) Kim, K. K.; Hsu, A.; Jia, X.; Kim, S. M.; Shi, Y.; Hofmann, M.; Nezich, D.; Rodriguez-Nieva, J. F.; Dresselhaus, M.; Palacios, T.; Kong, J. Synthesis of Monolayer Hexagonal Boron Nitride on Cu Foil Using Chemical Vapor Deposition. *Nano Lett* **2012**, *12* (1), 161–166. https://doi.org/10.1021/nl203249a.

(6) Lin, W.-H.; Brar, V. W.; Jariwala, D.; Sherrott, M. C.; Tseng, W.-S.; Wu, C.-I.; Yeh, N.-C.; Atwater, H. A. Atomic-Scale Structural and Chemical Characterization of Hexagonal Boron Nitride Layers Synthesized at the Wafer-Scale with Monolayer Thickness Control. *Chemistry of Materials* **2017**, *29* (11), 4700–4707. https://doi.org/10.1021/acs.chemmater.7b00183.

(7) Khan, M. H.; Liu, H. K.; Sun, X.; Yamauchi, Y.; Bando, Y.; Golberg, D.; Huang, Z. Few-Atomic-Layered Hexagonal Boron Nitride: CVD Growth, Characterization, and Applications. *Materials Today* **2017**, *20* (10), 611–628. https://doi.org/https://doi.org/10.1016/j.mattod.2017.04.027.

(8) Kidambi, P. R.; Blume, R.; Kling, J.; Wagner, J. B.; Baehtz, C.; Weatherup, R. S.; Schloegl, R.; Bayer, B. C.; Hofmann, S. In Situ Observations during Chemical Vapor Deposition of Hexagonal Boron Nitride on Polycrystalline Copper. *Chemistry of Materials* **2014**, *26* (22), 6380–6392. https://doi.org/10.1021/cm502603n.

(9) Moehring, N. K.; Chaturvedi, P.; Cheng, P.; Ko, W.; Li, A.-P.; Boutilier, M. S. H.; Kidambi, P. R. Kinetic Control of Angstrom-Scale Porosity in 2D Lattices for Direct Scalable Synthesis of Atomically Thin Proton Exchange Membranes. *ACS Nano* **2022**, *16* (10), 16003–16018. https://doi.org/10.1021/acsnano.2c03730.

(10) Cheng, P.; Kelly, M. M.; Moehring, N. K.; Ko, W.; Li, A.-P.; Idrobo, J. C.; Boutilier, M. S. H.; Kidambi, P. R. Facile Size-Selective Defect Sealing in Large-Area Atomically Thin Graphene Membranes for Sub-Nanometer Scale Separations. *Nano Lett* **2020**, *20* (8), 5951–5959.

(11) Cheng, P.; Moehring, N. K.; Idrobo, J. C.; Ivanov, I. N.; Kidambi, P. R. Scalable Synthesis of Nanoporous Atomically Thin Graphene Membranes for Dialysis and Molecular Separations via Facile Isopropanol-Assisted Hot Lamination. *Nanoscale* **2021**, *13* (5), 2825–2837.

(12) Chaturvedi, P.; Moehring, N. K.; Cheng, P.; Vlassiouk, I.; Boutilier, M. S. H.; Kidambi, P. R. Deconstructing Proton Transport through Atomically Thin Monolayer CVD Graphene Membranes. *Journal of Materials Chemistry A* **2022**. https://doi.org/10.1039/D2TA01737G.
(13) Cheng, P.; Fornasiero, F.; Jue, M. L.; Ko, W.; Li, A.-P.; Idrobo, J. C.; Boutilier, M. S. H.; Kidambi, P. R. Differences in Water and Vapor Transport through Angstrom-Scale Pores in Atomically Thin Membranes. *Nat Commun* **2022**, *13* (1), 6709.
(14) Hauwiller, M. R.; Ondry, J. C.; Alivisatos, A. P. Using Graphene Liquid Cell Transmission Electron Microscopy to Study in Situ Nanocrystal Etching. *JoVE* **2018**, No. 135, e57665.
(15) Park, J.; Elmlund, H.; Ercius, P.; Yuk, J. M.; Limmer, D. T.; Chen, Q.; Kim, K.; Han, S. H.; Weitz, D. A.; Zettl, A.; Alivisatos, A. P. 3D Structure of Individual Nanocrystals in Solution by Electron Microscopy. *Science (1979)* **2015**, *349* (6245), 290. https://doi.org/10.1126/science.aab1343.
(16) Regan, W.; Alem, N.; Alemán, B.; Geng, B.; Girit, Ç.; Maserati, L.; Wang, F.; Crommie, M.; Zettl, A. A Direct Transfer of Layer-Area Graphene. *Appl Phys Lett* **2010**, *96* (11), 113102. https://doi.org/10.1063/1.3337091.
(17) O'Hern, S. C.; Jang, D.; Bose, S.; Idrobo, J.-C.; Song, Y.; Laoui, T.; Kong, J.; Karnik, R. Nanofiltration across Defect-Sealed Nanoporous Monolayer Graphene. *Nano Lett* **2015**, *15* (5), 3254–3260. https://doi.org/10.1021/acs.nanolett.5b00456.
(18) O'Hern, S. C.; Boutilier, M. S. H.; Idrobo, J.-C.; Song, Y.; Kong, J.; Laoui, T.; Atieh, M.; Karnik, R. Selective Ionic Transport through Tunable Subnanometer Pores in Single-Layer Graphene Membranes. *Nano Lett* **2014**, *14* (3), 1234–1241. https://doi.org/10.1021/nl404118f.
(19) Cheng, P.; Kelly, M. M.; Moehring, N. K.; Ko, W.; Li, A.-P.; Idrobo, J. C.; Boutilier, M. S. H.; Kidambi, P. R. Facile Size-Selective Defect Sealing in Large-Area Atomically Thin Graphene Membranes for Sub-Nanometer Scale Separations. *Nano Lett* **2020**, *20* (8), 5951–5959. https://doi.org/10.1021/acs.nanolett.0c01934.
(20) Kidambi, P. R.; Jang, D.; Idrobo, J.-C.; Boutilier, M. S. H.; Wang, L.; Kong, J.; Karnik, R. Nanoporous Atomically Thin Graphene Membranes for Desalting and Dialysis Applications. *Advanced Materials* **2017**, *29* (33), 1700277. https://doi.org/10.1002/adma.201700277.
(21) Cheng, P.; Fornasiero, F.; Jue, M. L.; Ko, W.; Li, A.-P.; Idrobo, J. C.; Boutilier, M. S. H.; Kidambi, P. R. Differences in Water and Vapor Transport through Angstrom-Scale Pores in Atomically Thin Membranes. *Nat Commun* **2022**, *13* (1), 6709. https://doi.org/10.1038/s41467-022-34172-1.
(22) Cheng, P.; Ferrell, N.; Hus, S. M.; Moehring, N. K.; Coupin, M. J.; Warner, J.; Li, A.-P.; Fissell, W. H.; Kidambi, P. R. Protein-Enabled Size-Selective Defect-Sealing of Atomically Thin 2D Membranes for Dialysis and Nanoscale Separations. *Nano Lett* **2025**, *25* (1), 193–203. https://doi.org/10.1021/acs.nanolett.4c04706.
(23) Stehle, Y.; Meyer, H. M.; Unocic, R. R.; Kidder, M.; Polizos, G.; Datskos, P. G.; Jackson, R.; Smirnov, S. N.; Vlassiouk, I. V. Synthesis of Hexagonal Boron Nitride Monolayer: Control of Nucleation and Crystal Morphology. *Chemistry of Materials* **2015**, *27* (23), 8041–8047. https://doi.org/10.1021/acs.chemmater.5b03607.
(24) Tauc, J. Absorption Edge and Internal Electric Fields in Amorphous Semiconductors. *Materials Research Bulletin* **1970**, *5* (8), 721–729.

(25) Tauc, J.; Grigorovici, R.; Vancu, A. Optical Properties and Electronic Structure of Amorphous Germanium. *physica status solidi (b)* **1966**, *15* (2), 627–637.
(26) Shi, Y.; Hamsen, C.; Jia, X.; Kim, K. K.; Reina, A.; Hofmann, M.; Hsu, A. L.; Zhang, K.; Li, H.; Juang, Z.-Y.; Dresselhaus, Mildred. S.; Li, L.-J.; Kong, J. Synthesis of Few-Layer Hexagonal Boron Nitride Thin Film by Chemical Vapor Deposition. *Nano Lett* **2010**, *10* (10), 4134–4139. https://doi.org/10.1021/nl1023707.
(27) Kim, G.; Jang, A.-R.; Jeong, H. Y.; Lee, Z.; Kang, D. J.; Shin, H. S. Growth of High-Crystalline, Single-Layer Hexagonal Boron Nitride on Recyclable Platinum Foil. *Nano Lett* **2013**, *13* (4), 1834–1839. https://doi.org/10.1021/nl400559s.
(28) Gao, Y.; Ren, W.; Ma, T.; Liu, Z.; Zhang, Y.; Liu, W.-B.; Ma, L.-P.; Ma, X.; Cheng, H.-M. Repeated and Controlled Growth of Monolayer, Bilayer and Few-Layer Hexagonal Boron Nitride on Pt Foils. *ACS Nano* **2013**, *7* (6), 5199–5206. https://doi.org/10.1021/nn4009356.
(29) Watanabe, K.; Taniguchi, T.; Kanda, H. Direct-Bandgap Properties and Evidence for Ultraviolet Lasing of Hexagonal Boron Nitride Single Crystal. *Nat Mater* **2004**, *3* (6), 404–409. https://doi.org/10.1038/nmat1134.
(30) Elias, C.; Valvin, P.; Pelini, T.; Summerfield, A.; Mellor, C. J.; Cheng, T. S.; Eaves, L.; Foxon, C. T.; Beton, P. H.; Novikov, S. V; Gil, B.; Cassabois, G. Direct Band-Gap Crossover in Epitaxial Monolayer Boron Nitride. *Nat Commun* **2019**, *10* (1), 2639. https://doi.org/10.1038/s41467-019-10610-5.
(31) Gorbachev, R. V; Riaz, I.; Nair, R. R.; Jalil, R.; Britnell, L.; Belle, B. D.; Hill, E. W.; Novoselov, K. S.; Watanabe, K.; Taniguchi, T.; Geim, A. K.; Blake, P. Hunting for Monolayer Boron Nitride: Optical and Raman Signatures. *Small* **2011**, *7* (4), 465–468. https://doi.org/10.1002/smll.201001628.
(32) Vlassiouk, I.; Smirnov, S.; Puretzky, A.; Olunloyo, O.; Geohegan, D. B.; Dyck, O.; Lupini, A. R.; Unocic, R. R.; Meyer III, H. M.; Xiao, K. Armor for Steel: Facile Synthesis of Hexagonal Boron Nitride Films on Various Substrates. *Adv Mater Interfaces* **2024**, *11* (1), 2300704.
(33) Kidambi, P. R.; Mariappan, D. D.; Dee, N. T.; Vyatskikh, A.; Zhang, S.; Karnik, R.; Hart, A. J. A Scalable Route to Nanoporous Large-Area Atomically Thin Graphene Membranes by Roll-to-Roll Chemical Vapor Deposition and Polymer Support Casting. *ACS Appl Mater Interfaces* **2018**, *10* (12), 10369–10378. https://doi.org/10.1021/acsami.8b00846.
(34) Kidambi, P. R.; Nguyen, G. D.; Zhang, S.; Chen, Q.; Kong, J.; Warner, J.; Li, A.-P.; Karnik, R. Facile Fabrication of Large-Area Atomically Thin Membranes by Direct Synthesis of Graphene with Nanoscale Porosity. *Advanced Materials* **2018**, *30* (49), 1804977. https://doi.org/10.1002/adma.201804977.
(35) Cheng, P.; Ferrell, N.; Öberg, C. M.; Buchsbaum, S. F.; Jue, M. L.; Park, S. J.; Wang, D.; Roy, S.; Fornasiero, F.; Fissell, W. H.; Kidambi, P. R. High-Performance Hemofiltration via Molecular Sieving and Ultra-Low Friction in Carbon Nanotube Capillary Membranes. *Adv Funct Mater* **2023**, *33* (50), 2304672. https://doi.org/https://doi.org/10.1002/adfm.202304672.
(36) Kidambi, P. R.; Terry, R. A.; Wang, L.; Boutilier, M. S. H.; Jang, D.; Kong, J.; Karnik, R. Assessment and Control of the Impermeability of Graphene for Atomically Thin Membranes and Barriers. *Nanoscale* **2017**, *9* (24), 8496–8507. https://doi.org/10.1039/C7NR01921A.

(37) Kidambi, P. R.; Jang, D.; Idrobo, J.-C.; Boutilier, M. S. H.; Wang, L.; Kong, J.; Karnik, R. Nanoporous Atomically Thin Graphene Membranes for Desalting and Dialysis Applications. *Advanced Materials* **2017**, *29* (33), 1700277. https://doi.org/10.1002/adma.201700277.
(38) Ohlson, M.; Sörensson, J.; Haraldsson, B. Glomerular Size and Charge Selectivity in the Rat as Revealed by FITC-Ficoll and Albumin. *American Journal of Physiology-Renal Physiology* **2000**, *279* (1), F84–F91.
(39) Wang, D.; Ferrell, N. In Vitro Models to Evaluate Molecular Permeability of the Kidney Filtration Barrier BT - Kidney Research: Experimental Protocols; Hewitson, T. D., Toussaint, N. D., Smith, E. R., Eds.; Springer US: New York, NY, 2023; pp 41–53. https://doi.org/10.1007/978-1-0716-3179-9_4.
(40) Ng, M. L.; Shavorskiy, A.; Rameshan, C.; Mikkelsen, A.; Lundgren, E.; Preobrajenski, A.; Bluhm, H. Reversible Modification of the Structural and Electronic Properties of a Boron Nitride Monolayer by CO Intercalation. *ChemPhysChem* **2015**, *16* (5), 923–927. https://doi.org/https://doi.org/10.1002/cphc.201500031.
(41) Tanuma, S.; Powell, C. J.; Penn, D. R. Calculations of Electron Inelastic Mean Free Paths. V. Data for 14 Organic Compounds over the 50–2000 EV Range. *Surface and Interface Analysis* **1994**, *21* (3), 165–176. https://doi.org/https://doi.org/10.1002/sia.740210302.
(42) Yeh, J. J.; Lindau, I. Atomic Subshell Photoionization Cross Sections and Asymmetry Parameters: $1 \leqslant Z \leqslant 103$. *Atomic Data and Nuclear Data Tables* **1985**, *32* (1), 1–155. https://doi.org/https://doi.org/10.1016/0092-640X(85)90016-6.
(43) Shard, A. G. Practical Guides for X-Ray Photoelectron Spectroscopy: Quantitative XPS. *Journal of Vacuum Science & Technology A* **2020**, *38* (4), 41201. https://doi.org/10.1116/1.5141395.
